# Supplementary material for: Spatiotemporal organization of membrane protein controls bacterial extracellular electron transfer
Source: Nat Commun. 2026 Feb 17;17:2855. doi: 10.1038/s41467-026-69655-y (PMC13021941; doi:10.1038/s41467-026-69655-y)
Supplement: Supplementary file 1 — Supplementary Information [file 41467_2026_69655_MOESM1_ESM.pdf]

Supplementary Information

Spatiotemporal organization of membrane protein controls bacterial extracellular electron transfer

Youngchan Park, Tianlei Yan, Zhiheng Zhao, Bing Fu, Muwen Yang, Farshid Salimijazi, Buz Barstow, Peng Chen*

* Correspondence author: pc252@cornell.edu

Table of Contents

1. Supplementary Methods 3
1.1 Bacterial strains and growth culture condition 3
1.2 Genetic engineering of *Shewanella oneidensis* MR-1 4
1.3 Western blot for protein intactness 5
1.4 Ferrozine assay for cellular iron oxidation/reduction activity 6
1.5 Wide-field fluorescence imaging on coverslips for single-molecule tracking and single-cell quantification of protein concentration 6
1.6 Cell sample preparation and fluorescence imaging on indium-tin-oxide (ITO) coated glass for real-time monitoring 8
1.7 Menaquinone extraction 9
1.8 Bulk-level photoelectrochemistry measurements 9
1.9 Integrated single-cell photoelectrochemical current measurement and fluorescence imaging 10
Supplementary Notes 10
1. Validating functionality and intactness of FP-tagged proteins (CymA, STC, and FccA) for robust molecular imaging 10
2. Determination of CymA^{PmC} punctum size and location in the cell using machine learning segmentation 12
3. Fluorescent protein tags in anaerobically grown cells can mature sufficiently by post air-exposure to render successful fluorescence imaging and there is no significant synthesis of new CymA proteins in the cell within the timescale of 4-5 h of our imaging experiment 14
4. Determination of lag time and transition time of CymA punctum formation in the cell by fitting with a sigmoidal function 15
5. Skewness provides quantitative analysis of temporal dynamics of punctum formation in single cells 16
6. No colocalization between CymA and outer- or inner-membrane EET proteins under anaerobic growth conditions with active EET 19
6.1 Tagging PmC to outer and inner membrane EET proteins (OmcA/MtrCAB, Dld-II, Ndh) and their spatial distributions in the cell do not show punctate features under anaerobic growth 19
6.2 No significant colocalization of CymA puncta with outer membrane extensions (OME) generation spots 20
7. Challenges in measuring photoanodic current for electron outflow 21
8. Reactive oxygen species (ROS) does not play a significant role in enhancing electron-uptake capabilities by cells in our study 22
9. Discussion about single-cell current measurement 23
10. Discussion about CymA expression levels and EET function 24
11. No significant difference in cellular concentrations of STC/FccA/OmcA for anaerobically grown cells that had formed puncta vs. those that had not 25
12. Analysis of diffusion dynamics of CymA in the cell to resolve its minimal number of diffusion states as well as the associated diffusion constants (*D*) and fractional populations (*A*) 26
12.1 Inverse transformation of confined displacement distribution (ITCDD) algorithm to deconvolute the cell confinement effect on the displacement length distribution obtained from single-molecule tracking 26
12.2 Determination of diffusion states of CymA in the cell from ITCDD-corrected CDF 27
13. Reconstruction of super-resolution images from single-molecule tracking trajectories 28
13.1 Reconstruction of single molecule tracking trajectories to super-resolution images sorted by displacement 28
13.2 CymA molecules either within or outside puncta have similar mobility 30
14. Kinetics of CymA protein moving out or into CymA puncta 31
14.1 Determination of escaping kinetics of CymA out of puncta 31
14.2 Determination of effective rate constant of forming puncta via equilibrium approximation 33
15. Additional supplementary figures 33

1. Supplementary Methods

1.1 Bacterial strains and growth culture condition

Shewanella oneidensis MR-1 (referred to as *Shewanella oneidensis* for brevity in this work) and *E. coli* WM3064 (developed by William Metcalf from UIUC, unpublished) were obtained from Buz Barstow at Cornell University. All other strains were constructed in the lab as described in Supplementary Methods 1.2 below. *S. oneidensis* was grown in 5 mL LB overnight at 30 °C for 20 h until OD_{600} of the cell culture reached ~1.0. Then, the overnight culture was spun down, washed with fresh LB media, and diluted to OD 0.1 for glycerol stock. *E. coli* WM3064 was grown in 5 mL LB supplemented with DAP (diaminopimelic acid) overnight at 37 °C for 16 h until OD_{600} of the cell culture reached ~2.0. Overnight culture was spun down, washed with fresh LB media, and diluted to OD 0.2 for glycerol stock.

Aerobic growth condition for imaging. *S. oneidensis* was inoculated from glycerol stock to LB medium and grown at 30 °C for 20 h. The overnight culture was diluted to 1:50 in 5 mL minimal medium (MM) supplemented with amino acids (GIBCO, cat. #: 11130051) and vitamins (GIBCO, cat. #: 11120052), further grown to OD_{600} of ~0.4. Amino acids act as a carbon source[1–3] After being grown, cells were collected and resuspended in 100 µL to be OD_{600} ~20. MM recipe for growing *S. oneidensis*[4,5] Dibasic sodium phosphate (Na_2HPO_4), 7 g/L; monobasic potassium phosphate (KH_2PO_4), 3 g/L; sodium chloride (NaCl), 0.5 g/L; ammonium chloride (NH_4Cl), 1 g/L; magnesium sulfate heptahydrate ($MgSO_4$-$7H_2O$), 1mM; calcium chloride ($CaCl_2$), 0.3 mM; 1X trace element solution (100X trace element solution: EDTA, 5 g; iron chloride hexahydrate ($FeCl_3$-$6H_2O$), 0.83 g; zinc chloride ($ZnCl_2$), 84 mg; copper chloride dihydrate ($CuCl_2$-$2H_2O$), 13 mg; cobalt chloride dihydrate ($CoCl_2$-$2H_2O$), 10 mg; boric acid (H_3BO_3), 10 mg; manganese chloride tetrahydrate ($MnCl_2$-$4H_2O$), 1.6 mg). The pH of the media was adjusted to pH 7.2 with sodium hydroxide (NaOH) and sterilized by autoclave.

Anaerobic growth condition for imaging. *S. oneidensis* was inoculated from glycerol stock to LB medium and grown at 30 °C for 20 h. The overnight culture was diluted to OD_{600} of ~0.1 in 10 mL of MM supplemented with an electron donor and acceptor. The 10 mL tube was purged with N_2 gas for 15 min and sealed tight with rubber septa. The cells in the sealed tube were grown at 30 °C for 2 days. Specifically, sodium lactate 20 mM or 12 mg iron (>99%, fine powder, catalog #: 12310, Sigma-Aldrich) was used as an electron donor; and ferric citrate 20 mM, ferric oxide 20 mM, or sodium fumarate 20 mM was used as an electron acceptor. When hydrogen gas was used as an electron donor, the medium was purged with 7% H_2 balanced with N_2 gas for 15 min. After being grown anaerobically, cells were collected and resuspended in 25 µL to be OD_{600} ~20 under aerobic conditions, during which the fluorescent proteins mature sufficiently for successful fluorescence imaging (Supplementary Methods 1.5; Supplementary Notes 3).

Supplementary Table 1 | Various conditions that *Shewanella oneidensis* cells were cultured.

Condition	Aerobic/ anaerobic	Electron donor	Electron acceptor	solubility of extracellular substrate	e^- flow direction	Mtr Pathway?	CymA is involved in EET?	Note
1	Aerobic	Amino acids	O_2			no	no	Denoted as AA/O_2 in Fig. 1
2	Anaerobic	Sodium lactate 20 mM	Ferric citrate 20 mM	Soluble	outflow	yes	yes	Denoted as Lac/$Fe^{3+}(aq)$ in Fig. 1
3	Anaerobic	7% H_2	Sodium fumarate 20 mM	Soluble	uptake	no	yes	Denoted as H_2/Fum in Fig. 1
4	Anaerobic	Sodium lactate 20 mM	Ferric oxide 20 mM	insoluble	outflow	yes	yes	Denoted as Lac/$Fe^{3+}(s)$ in Fig. 1

5	Anaerobic	Metal iron	Sodium fumarate 20 mM	insoluble	uptake	yes	yes	Denoted as Fe/Fum in Fig. 1
6	Anaerobic	NADH 0.1 mM	Ferric citrate 0.1 mM	soluble	outflow	yes	yes	For Ndh functionality test
7	Anaerobic	–480 mV or –280 mV (vs. Ag/AgCl)	Sodium fumarate 20 mM	insoluble	uptake	yes	yes	For imaging on ITO coated glass for real-time monitoring (Supplementary Methods 1.6)
8	Anaerobic	Sodium lactate 20 mM	+280 mV (vs. Ag/AgCl)	insoluble	outflow	yes	yes	For imaging on ITO coated glass for real-time monitoring (Supplementary Methods 1.6)

1.2 Genetic engineering of *Shewanella oneidensis* MR-1

To genetically tag a specific protein with a fluorescent protein (FP) at its chromosomal locus, biparental conjugation method was used for all genetic engineering procedures[6,7]. The proteins that were tagged in this study were all done as C-terminal fusions in their respective chromosome loci, and their C-terminals are all solvent-exposed based on the crystal structures[8–11] (or homology structure for CymA[12], Ndh and Dld-II[13,14]). Initial step involved the construction of a conjugation plasmid through Gibson assembly, incorporating three DNA fragments into the multicloning site of pRE118 (Addgene plasmid # 43830, carrying the kanamycin resistance gene and the *sacB* gene, which confers sensitivity to sucrose)[15]. These three fragments comprised a 1000 bp segment homologous to the upstream region of the target gene's stop codon (homologous region 1, HR1), another 1000 bp segment homologous to the downstream region of the target gene's stop codon (homologous region 2, HR2), and the fusion protein FP-FLAG positioned between HR1 and HR2. The addition of FLAG at the end facilitates potential western blot analysis. Subsequently, the constructed conjugation plasmid was introduced into the donor *E. coli* strain WM3064 via electroporation (18.0 kV/cm, Bio-Rad MicroPulser Electroporator). The successfully transformed donor strain, identified through screening for kanamycin resistance encoded in the original pRE118 plasmid, was then combined with *S. oneidensis* recipients at a 10:1 ratio and cultivated on an LB plate at 30 °C for 28 h. Initial crossover colonies were selected on a plate with 300 µg/mL kanamycin, as the recipient cells were expected to acquire kanamycin resistance from the integrated plasmid in their genome. The successful first crossover colonies were picked and streaked on a subsequent plate containing 10% w/v sucrose for the second crossover. The desired second crossover colonies should eliminate the *sacB* gene, leaving only the fluorescent protein gene intact, causing any undesired colonies still retaining the *sacB* gene to be eliminated in the presence of sucrose. The final colonies were subjected to PCR screening, and their identities were confirmed through Sanger DNA sequencing. For double-tagged strains, the second fluorescent tag was inserted into the genome sequentially using the same method described above, building upon the single-tagged strain. Specific gene knockouts followed a similar process, but the plasmid design omited the fluorescent tag fusion, containing only HR1 and HR2, which flanked the upstream and downstream regions of the entire target gene. Primers used in this study are listed in the Source Data.

Supplementary Table 2 | Strains used or constructed in this study

**Strains**	**Chromosomal modification**	**Reference or source**
Shewanella oneidensis MR-1	Wild-type, base strain	from Buz Barstow
Δ*cymA*	Δ*cymA* (SO4591)	This study
Δ*fccA*	Δ*fccA* (SO0970)	This study
Δ*stc*	Δ*stc* (SO2727)	This study

Δ*fccA*, Δ*stc*	Δ*fccA*, Δ*stc*	This study
Δ*mtrB*	Δ*mtrB* (SO1776)	This study
Δ*mtrA*	Δ*mtrA* (SO1777)	This study
Δ*mtrC*	Δ*mtrC* (SO1778)	This study
Δ*omcA*	Δ*omcA* (SO1779)	This study
Δ*ndh*	Δ*ndh* (SO3517)	This study
Δ*dld-II*	Δ*dld-II* (SO1521)	This study
Δ*menB*	Δ*menB* (SO4739)	This study
Δ*menD*	Δ*menD* (SO4573)	This study
$CymA^{PmC}$	*cymA-PAmCherry1-FLAG*	This study
$CymA^{GFP}$	*cymA-sfGFP-FLAG*	This study
$CymA^{GFP}$, Δ*fccA*	*cymA-sfGFP-FLAG, ΔfccA*	This study
$FccA^{PmC}$	*fccA-PAmCherry1-FLAG*	This study
$FccA^{PmC}$, $CymA^{GFP}$,	*fccA-PAmCherry1-FLAG, cymA-sfGFP-FLAG*	This study
$FccA^{GFP}$, Δ*stc*	*fccA-PAmCherry1-FLAG,* Δstc	This study
STC^{PmC}	*stc-PAmCherry1-FLAG*	This study
STC^{PmC}, $CymA^{GFP}$	*stc-PAmCherry1-FLAG, cymA-sfGFP-FLAG*	This study
STC^{PmC}, Δ*fccA*	*stc-PAmCherry1-FLAG, ΔfccA*	This study
$MtrB^{PmC}$	*mtrB-PAmCherry1-FLAG*	This study
$MtrA^{PmC}$	*mtrA-PAmCherry1-FLAG*	This study
$MtrC^{PmC}$	*mtrC-PAmCherry1-FLAG*	This study
$OmcA^{PmC}$	*omcA-PAmCherry1-FLAG*	This study
Ndh^{PmC}	*ndh-PAmCherry1-FLAG*	This study
$Dld\text{-}II^{PmC}$	*Dld-II-PAmCherry1-FLAG*	This study
E. coli WM3064	Wild-type, donor strain for conjugation	from Buz Barstow (developed by William Metcalf in UIUC)

1.3 Western blot for protein intactness

Appropriate strains were inoculated and grown in LB medium at 30 °C for 20 h. The overnight culture was diluted to OD_{600} of ~0.1 in MM supplemented with amino acids and vitamin for aerobic culture or sodium lactate and ferric citrate for anaerobic culture (Supplementary Methods 1.1 for detailed growth conditions and procedures). After incubation, cells were washed with MM and collected and suspended in 1 mL to be OD_{600} ~ 1.0 (aerobically cultured cells have to be diluted and anaerobically cultured cells have to be concentrated). 1 mL of cultures of identical OD were centrifuged for 5 min (1600 g) and supernatant was removed. The resulting cell pellets were gently re-suspended in 95 µL 2X Laemmli Sample Buffer (BIORAD, cat. #: 1610737) and 2.5 µL of beta-mercaptoethanol (Sigma-Aldrich, cat. #: M6250) and 2.5 µL of Protease Inhibitor Cocktail (Promega, cat. #: G6521) were added to the suspension, followed by sonication for 15 min. After sonication, 10 µL was electrophoresed on a 4–20% polyacrylamide gel (Bio-Rad, cat. #: 4561096) in MES buffer (pH = 7.3) for 130 min at 120 V. Then, proteins were transferred onto a PVDF membrane (GE Healthcare Life Sciences) for 70 min at 100 V using a Mini Trans-Blot Cell (Bio-Rad, USA). The membrane was blocked with 4% blocking reagent (GE Healthcare Life Sciences, Amersham ECL Prime blocking reagent) dissolved in PBST (0.1% Tween-20 in 1X PBS) for 1 h and incubated overnight at 4 °C with the primary antibodies (1:10,000), rabbit anti-FLAG antibody (Rockland Immunochemical, cat. #: RL600- 401-383S) after washing out blocking agent thoroughly. After overnight incubation, washing the membrane with 1X PBST thoroughly. Then the membrane was incubated with the secondary antibodies (1:20,000), goat-derived Horseradish Peroxidase–conjugated Fab fragment anti-rabbit secondary antibody (Rockland Immunochemical, cat. #: RL811-1302), for 1 h. The membrane was washed five times with 1X PBST and then developed using the Pierce ECL 2 Western Blotting substrate (Fisher Scientific, cat. #: PI80196) and subsequently documented using GelDoc Gel imaging system (Bio-Rad, USA). The band intensities were quantified using ImageJ.

1.4 Ferrozine assay for cellular iron oxidation/reduction activity

For functionality test, appropriate strains were inoculated from glycerol stock and grown in LB medium at 30 °C for 20 h. The overnight culture was diluted to OD_{600} of ~0.1 in 5 mL MM supplemented with sodium lactate and ferric citrate (NADH and ferric citrate for Ndh^{PmC}), followed by culturing anaerobically for 2 days (Supplementary Methods 1.1 for detailed growth conditions and procedures). The same sample without cells was also prepared and kept anaerobically for 2 days as control. After incubation, HCl was added to the sample to dissolve solid ferrous citrate (creamy off-white precipitation), kept the solution pH 5-7. Then, 10 µL of the sample was added to 2 mL of 100 mM ferrozine solution and mixed well. Ferrozine forms a stable magenta-colored solution with the ferrous ion[16]. The absorbance of the samples was measured at 562 nm.

For monitoring cellular iron oxidation/reduction as a function of time, WT strains were inoculated from glycerol stock and grown in LB medium at 30 °C for 20 h. The overnight culture was diluted to OD_{600} of ~0.1 in 5 mL MM supplemented with sodium lactate and ferric citrate for iron reduction activity detection or metal iron and sodium fumarate for iron oxidation activity detection, followed by anaerobic culture. For iron reduction activity monitoring, the procedure is same as described above. For iron oxidation activity monitoring, the sample was centrifugated to remove solid metallic iron after anaerobic culture. Then, 10 µL of the sample was added to ferrozine solution and measured the absorbance as described above.

1.5 Wide-field fluorescence imaging on coverslips for single-molecule tracking and single-cell quantification of protein concentration

Microscope setup. For fluorescence imaging of cells on coverslips, fluorescence signals were captured using a 60× oil immersion objective (Olympus PlanApo N 60× oil 1.45 TIRFM UIS 2) on an Olympus IX71 inverted microscope equipped with transmission optics (Supplementary Fig. 1a). An EMCCD camera (Andor Technology, DU-897E-CSO-#BV, pixel size 16×16 μm^2) served as the detector. The image was further magnified by a 1.6× magnification changer in the microscope and a 1.2× C-mount adaptor (Spot Diagnostic Instruments, DD12BXC), resulting in a final image pixel size of 135.4^2 nm^2. Coaxially aligned continuous wave lasers (405 nm, CrystaLaser, DL405-100; 488 nm, Coherent, Sapphire 488-100 CW CDRH; 561 nm, Coherent, Sapphire 561-100 CW CDRH) passed through dichroic filters (Chroma, T510lpxru and T425lpxr) and a broadband quarter waveplate to be changed from linear polarized to circular polarized. The lasers, expanded four times by an achromatic lens pair and focused (40 cm lens) at the back focal plane of the objective, were then reflected toward the objective by a three-band dichroic filter (Chroma, ZT405/488/561rpc) inside turret cube. Epi-illumination was inclined approximately 60° from the optical axis of the objective to minimize background from the medium. In the emission detection path, a bandpass filter (Semrock, FF01-617/73) was used for detecting the red fluorescence from photoactivated PAmCherry1, while a bandpass filter (Chroma, ET525/50m) was used for detecting the green fluorescence from sfGFP or SynaptoGreen, All images were captured in stroboscopic mode with integration time of 4 ms and a time-lag of 60 ms between images; the laser exposure was controlled by an AOTF and synchronized with the EMCCD exposure, as we reported previously[7,17,18].

Sample preparation. To prepare *S. oneidensis* cells for imaging, the bacterial cells were sandwiched between a borosilicate coverslip and an agarose pad with double-sided tapes around it as spacer from a top glass slide (Supplementary Fig. 1b), following our previously described method[7,18,19]. Briefly, 50 µL of 100 nm gold nanoparticles (Ted Pella, Inc., cat. #: 15708-9) in a 1:1 water-ethanol solution were drop-casted onto a clean coverslip to serve as position markers for drift correction. Subsequently, a 3% w/v gel pad was created by heating 1 mL of MM containing 0.03 g of agarose and drop-casting the solution onto a glass slide with Parafilm spacers, which was then pressed by another glass slide. Once the gel was solidified, 0.5-1 µL concentrated cells (OD_{600} ~20, see Supplementary Methods 1.1) were applied on top of the agarose gel pad on a glass slide. The coverslip with gold nanoparticles was then positioned on the gel pad, effectively spreading and immobilizing the cells. To prevent gel drying and medium evaporation, the coverslip edges were sealed with epoxy (Supplementary Fig. 1b).

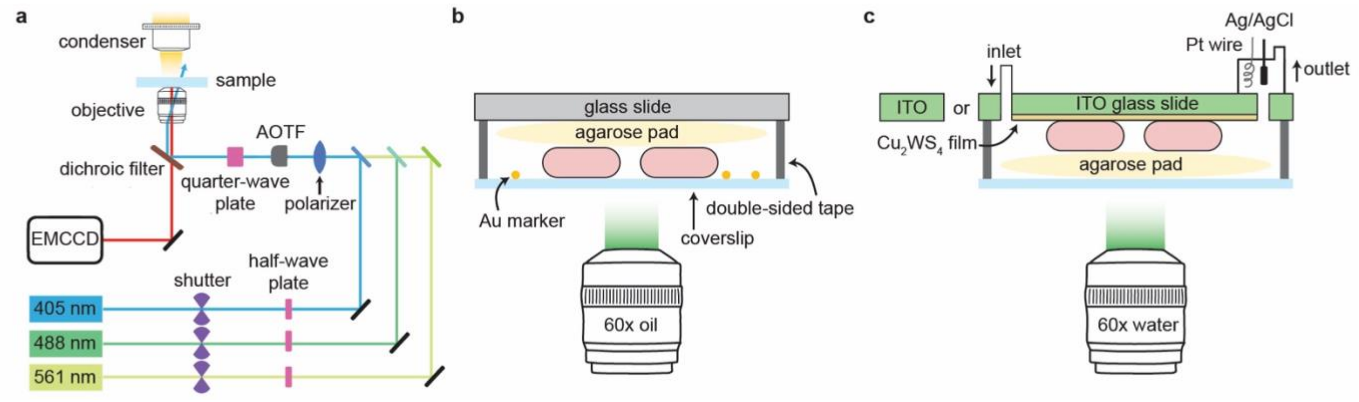

Supplementary Fig. 1 | Imaging setup and configurations for single-cell imaging. a, Optical setup for overall fluorescence imaging. AOTF: acoustic optical tunable filter. **b,** Sample configuration for imaging on coverslip for single-molecule tracking and single-cell quantification of protein concentration. **c,** Microfluidic cell configuration for (photo)electrochemical fluorescence imaging on a thin-film Cu_2WS_4 deposited on an ITO-coated glass or bare ITO glass.

Wide-field fluorescence imaging on coverslip. To observe only protein location or colocalization between two proteins through fluorescence imaging, the following procedures were performed. For PmC-tagged strain imaging (CymA^{PmC}, STC^{PmC}, FccA^{PmC}, MtrA^{PmC}, MtrC^{PmC}, OmcA^{PmC}, Dld-II^{PmC}, Ndh^{PmC}), cells were first illuminated by ~5 W/cm^2 405 nm for a duration of 1 min to activate all PAmCherry1 fusion proteins, and then excited by ~3 kW/cm^2 561 nm and imaged with EMCCD. For super folder GFP-tagged strain imaging (CymA^{GFP}, CymA^{GFP}+ Δ*fccA*), cells were directly excited with ~3 kW/cm^2 488 nm laser. For doubly FP-tagged strain imaging (CymA^{GFP}+STC^{PmC}, CymA^{GFP}+FccA^{PmC}), GFP-tagged proteins were firstly imaged with 488 nm to avoid degradation of GFP by blue light (405 nm activation light for PmC)[20] as described above and were subsequently completely photobleached. Then PmC tagged proteins were activated with 405 nm and imaged with 561 nm as described. For SynaptoGreen-stained CymA^{PmC} strain imaging, PmC-tagged protein was firstly activated by 405 nm laser and then imaged with 561 nm laser as described above. Then, cells were stained with SynaptoGreen and directly excited with ~300 W/cm^2 488 nm laser for imaging.

Single-molecule tracking (SMT) and single-cell quantification of protein concentration (SCQPC) using photoactivatable fluorescence protein. Single-molecule tracking (SMT) employing stroboscopic imaging was conducted with a short laser pulse duration controlled by an acousto-optic tunable filter (AOTF, AA, AOTFnC-400.650-TN) as described previously[7,17–19]. In the SMT process, cells were initially exposed to a 405 nm laser (2-5 W/cm^2, the intensity was adjusted depending on protein) for 20 ms to photoactivate a single PAmCherry1 molecule (or none). Subsequently, a series of 30 pulses from a 561 nm laser (~3 kW/cm^2) in epi-illumination mode, with a pulse duration of 4 ms (T_{int}) and time lag of 60 ms (T_{tl}), were employed to excite the photoactivated PAmCherry1. The resulting fluorescence from a single molecule was imaged by an EMCCD camera with 250 EM gain, synchronized with the 561 nm laser pulses. This imaging procedure was iterated for 500 cycles for each field of view. The effective localization uncertainty of our single-molecule imaging is ~40 nm. SCQPC was performed after SMT on the same cells so single-molecule intensity could be directly obtained from SMT process, similarly as we reported previously[19,7,17,18]. Cells were illuminated with the 405 nm laser (~5 W/cm^2) for 1 min to photoactivated all remaining FP molecules, followed by 561 nm laser illumination for 2000 frames with the same laser power density and exposure time as in the SMT step to quantify the number of remaining PAmCherry1 molecules, whereas EM gain was changed to 100 to avoid camera saturation (this EM gain difference was taken into account via linear extrapolation in calculating the protein copy numbers). This 405-illumination and 561-excitation sequence was repeated twice to ensure all PAmCherry1 molecules had been photoactivated and imaged. The total protein copy number in each cell was estimated by taking into account the photoactivation efficiency of PAmCherry1 (50%)[21].

We performed the same SMT+SCQPC imaging process using a wild-type *S. oneidensis* strain that does not contain any fluorescent protein tag as a control experiment. There are some false positive single-molecule signals during SMT and a slight fluorescence level during SCQPC, amounting together to 52 ± 36 molecules on average per cell (Supplementary Fig. 2). Compared with the copy number of CymA^{PmC} (~4.0×10^3 in copy number on average, see Supplementary Table 4for details), these false detections are less than 2%, and thus negligible.

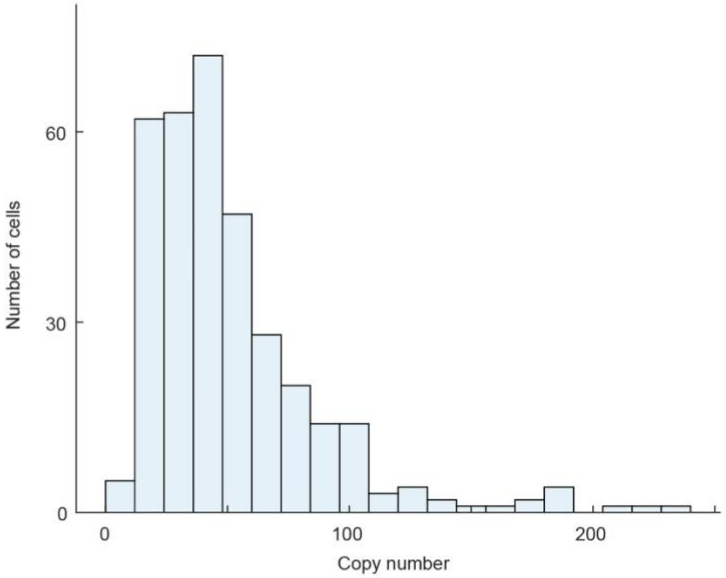

Supplementary Fig. 2 | Control SMT+SCQPC imaging for false detection of protein copy numbers in single cells. Copy number is obtained from the wild-type *S. oneidensis* that contains no fluorescent protein tags. 345 cells were counted. Mean ± s.d. = 52 ± 36. Source data are provided as a Source Data file.

A custom-written MATLAB code called iQPALM (Image-based Quantitative Photo-Activated Localization Microscopy) was used to extract cell contours, subsequently fitting them to a hemisphere-capped-rod model to ascertain cell volume[17,22]. The contours of cells that overlap with each other were not extracted and analyzed. Protein concentration was calculated as the total cellular copy number divided by the corresponding cell volume. Additionally, the same set of cell contours were applied to define the region for integrating fluorescence intensity pixels, facilitating the determination of the overall cellular copy number.

1.6 Cell sample preparation and fluorescence imaging on indium-tin-oxide (ITO) coated glass for real-time monitoring

Sample preparation. Overall procedure is similar to sample preparation for fluorescence imaging (Supplementary Methods 1.5) except that the bacterial cells were sandwiched between ITO-coated glass slide and an agarose pad. 0.5-1 μL of aerobically grown cells (OD_{600} ~ 20, $\text{CymA}^{\text{PAmC1}}$ strain) were deposited on ITO glass to have direct contact with the ITO electrode and an agarose pad with a thickness of ~100 μm was placed on top to immobilize the cells. The whole construct was then assembled into the electrochemical microfluidic cell (~100 μm high and ~5 mm wide) using double-sided tapes sandwiched between a microscope coverslip and the ITO electrode (Supplementary Fig. 1c). After the assembled microfluidic cell was completely sealed with epoxy and dried, the minimum medium purged with N_2 was introduced into the microfluidic cell by a syringe pump. The medium contained fumarate when negative potential was applied. The ITO electrode serves as the working electrode, the electrochemical potential of which is controlled by a potentiostat (CH Instrument, CHI1200a). A platinum wire and an Ag/AgCl (3 M NaCl) electrode (BASi, MW-2030) were used as the counter and reference electrode, respectively, and were placed in a liquid chamber about ~3 cm downstream.

Wide-field fluorescence imaging while applying potential. The same microscope setup was used for fluorescence imaging except for a 60× water immersion objective (Olympus, UPlanSApo 60× NA 1.20 water UIS 2), which has a longer working distance to image through the microfluidic channel to see the cells in contact with ITO (Supplementary Fig. 1c). Cells were illuminated with the 405 nm laser (~5 W/cm^2) for 2 min to photoactivate all FP molecules, followed by 561 nm laser (~1.5 kW/cm^2) illumination for 3 frames with 15 ms exposure time and EM gain 100 to avoid camera saturation. After an electrochemical potential of –0.48 V, –0.28 V or +0.60 V (vs Ag/AgCl) was applied and held constantly, fluorescence images were captured every 15 – 30 min for 6 h for cells with cathodic potential where electrons are uptaken by cells and every 3 h for 15 h for cells with anodic potential where electrons are outflowing from cells.

1.7 Menaquinone extraction

Menaquinone was extracted from *S. oneidensis* using lysozyme-chloroform-methanol extraction method as reported[23]. Briefly, lysozyme was used to lysate the bacterial cells and chloroform-methanol (2:1 v/v) for extraction. First, 50 mL or 500 mL of cells were cultured aerobically (OD_{600} ~ 1) or anaerobically (OD_{600} ~0.1) to have 100 mg of wet cellular mass. Then, cells were washed twice with MM and suspended in 5 mL of MM with 5 mg of lysozyme, followed by incubating at 37 °C for 1 h. After centrifugation to remove media, the cells were washed with 500 µL of methanol to remove water. Then, 1 mL of chloroform-methanol (2:1 v/v) solution was added and shaken for 1 min. this chloroform-methanol extraction was repeated three times. All liquid including methanol was collected and dried with rotary evaporator at 40 °C and dissolved in 50 µL of chloroform-methanol solution for thin layer chromatography (TLC). TLC was performed using silica gel HF254 (20 × 20 cm, Sigma Aldrich cat#: 100390) and the extracts were developed with hexane/diethyl ether (85:15, v/v) solvent. The menaquinone migrates at retention factor (Rf) ~ 0.7[23,24].

1.8 Bulk-level photoelectrochemistry measurements

Cu_2WS_4 synthesis. Cu_2WS_4 particles were synthesized via a hydrothermal process, following a protocol slightly revised from the method developed by Li et al.[25], as we reported previously[7]. Briefly, 0.0025 mol $Na_2WO_4\cdot 2H_2O$ (Sigma-Aldrich), 0.005 mol CuCl (Sigma-Aldrich), and 0.0125 mol thioacetamide (Sigma-Aldrich) were mixed in the solution consisting of 15 mL deionized water and 15 mL ethanol. The mixture was stirred for 20 min to achieve a homogeneous suspension. Then, the resulting suspension was transferred to a 50 mL Teflon-lined stainless-steel autoclave, sealed, and heated at 200 °C for 120 h. Following cooling, the product was centrifuged and washed with deionized water and ethanol multiple times. These Cu_2WS_4 particles are truncated bipyramid shapes with sizes varying between 1 µm and 10 µm; they are also photocathode materials with visible absorption and a conduction band edge (E_{CB}) appropriate for reducing protons and other common cellular redox proteins (Supplementary Fig. 24). As we reported previously[7], Cu_2WS_4 thin-film photoelectrodes alone show clear steady-state photocathodic currents from proton reduction with 405-nm light excitation and an applied appropriate potential (stable for >3 h in N_2).

Bulk-level photoelectrochemical measurements. Bulk-level photoelectrochemistry experiments were performed using Cu_2WS_4 thin films deposited on ITO-coated glass slides (Delta Technologies, CB-50IN-0105) with and without anaerobically pre-grown wild-type *S. oneidensis* cells under N_2, similarly as we reported previously in measuring bulk photoelectrochemistry of bacterial cells[7]. Cu_2WS_4 particle suspensions in water (1 mg/mL) were sonicated for ~1 h to disperse any precipitates, and 200 µL of such particle suspension was then drop-casted onto an ITO electrode. The resulting semiconductor film deposited on ITO was annealed with a furnace (Thermo Scientific Lindberg/Blue M) at 80 °C overnight. To perform control experiments without the cells, the annealed electrodes were mounted in a homemade chamber (2 cm diameter × 2 cm height cylindrical shape) filled with 1 mL of MM thoroughly purged with N_2. The whole constructs were illuminated by an expanded 405 nm laser beam (405 nm, Coherent, OBIS 1265575) with a diameter of ~2.5 cm and a power density of ~1 mW/cm^2. The 405 nm laser was kept on for a duration of 15 s and off for a duration of 10 s. The applied potential on the Cu_2WS_4 was held constant at –0.2 V (vs

Ag/AgCl). After conducting the control experiment, a few drops of a high-density suspension of the concentrated cells (OD_{600}~20) were deposited onto the semiconductor film. The photocurrent was then measured in the same manner. All photoelectrochemical measurements were performed using a potentiostat (CH Instrument, CHI1200a) in a three-electrode configuration. The Cu_2WS_4-deposited ITO electrodes served as the working electrode, a Pt wire (BASi, MW-4130) as the counter electrode, and an Ag/AgCl electrode (BASi, MW-2030) as the reference electrode.

1.9 Integrated single-cell photoelectrochemical current measurement and fluorescence imaging

Microscope setup. Photoelectrochemical current measurement and fluorescence imaging at the single-cell level utilize an optical configuration based on an inverted Olympus IX71 microscope equipped with a 60× water immersion objective (Olympus, UPlanSApo 60× NA 1.20 water UIS 2), similar to what we reported previously[7]. This setup is complemented by a three-electrode photoelectrochemical microfluidic cell (Supplementary Fig. 1c) and an EMCCD camera (Andor Technology, DU-897D-CSO-#BV, pixel size 16×16 μm^2), serving as the detector[7]. Coaxially aligned continuous wave lasers (405 nm, Coherent, OBIS 1265575; 488 nm, CrystaLser DL488-050) passed through dichroic filters (Thorlab, DMLP425) and a broadband quarter waveplate to convert linear polarized to circular polarized. A suitable combination of a flip lens (Thorlabs, N-BK7 Plano-Convex Lens) mounted on an XYZ translation stage (Thorlabs, PT3-1, 1/4-20 Taps) placed at the back port of the microscope enables switching between wide-field fluorescence imaging and the focused laser illumination for single-cell photocurrent measurements. The lasers were then directed toward the objective by dichroic filter (Chroma, ZT405rdc and ZT375/488/532/635rpc) inside turret cube. In the emission path, a bandpass filter (Semrock, FF01-531/40) was employed for detecting green fluorescence from sfGFP.

Single-cell photoelectrochemical current measurement and fluorescence imaging. Samples were prepared as in electrochemical fluorescence imaging (Supplementary Methods 1.5) except that the cells were deposited on a Cu_2WS_4 thin-film on top of an ITO electrode, which serves as the working electrode, similarly as we reported for measuring other bacterial cells[7]. Cells were illuminated with the 488 nm laser (~5 W/cm^2) to excite and acquire sfGFP fluorescence images for 3000 frames with 30 ms exposure time and EM gain 300 to avoid camera saturation. Cell contours were identified from fluorescence images. The total protein copy number was determined by dividing whole-cell fluorescence by the single-molecule intensity derived from *S. oneidensis* $CymA^{sfGFP}$ at the same power density and EM gain.

Subsequently, the circularly polarized 405-nm laser, which excites the charge carriers in Cu_2WS_4, underwent enlargement via a beam expander, and focused by the objective to generate a laser spot. By adjusting the distance of the beam expander and slightly defocusing, the size of the laser spot at the imaging focal plane was adjusted to a diameter of ~3 μm, sufficient to cover an entire single cell. This laser spot could be positioned either on top of a cell (on-cell location) or near the cell (off-cell location) by moving the sample stage. Upon placing the laser spot at the desired location, chronoamperometric measurements at –0.2 V (vs. Ag/AgCl) were conducted during light-chopping, typically involving 15 s of on-period followed by 10 s of off-period for three cycles. To quantify the photocurrent, the currents for the 2-3 s durations on either side of the time of turning illumination on, (t_{on} – 3 s, t_{on} – 1 s) and (t_{on} + 1 s, t_{on} + 4 s), were fitted with two different linear functions. The difference in these two linear functions at t_{on} was taken as the photocurrent (i.e., i_{ph}) for this light on/off cycle. This method was detailed in our previous work[7].

Supplementary Notes

1. Validating functionality and intactness of FP-tagged proteins (CymA, STC, and FccA) for robust molecular imaging

Functionality of FP-tagged CymA, STC, and FccA. For CymA^{PmC}, we assessed its Fe(III) reduction ability through the Ferrozine assay on the *S. oneidensis* cells (Supplementary Methods 1.4)[16,26]. First, we confirmed that Fe(III) reduction does not occur in the absence of cells (Supplementary Fig. 3, column 1). To confirm CymA's reduction activity, we cultured wild-type (WT), FP-tagged (i.e., PmC- or GFP-tagged), and deletion strains anaerobically with lactate and ferric citrate. The resulting Fe(II) production was measured and plotted relative to that of WT strain (column 2-5). Both PmC- and GFP-tagged CymA strains showed comparable Fe(III) reduction activity to WT, while the Δc*ymA* deletion strain displayed a significant reduction in activity. Same assays were carried out for STC and FccA. Because STC and FccA share a role in electron shuttling between the inner membrane (IM) and outer membrane (OM)[27], single Δ*fccA* or Δ*stc* deletion strain retained the ability to reduce Fe(III) as much as WT, indicating they compensate each other's role effectively (column 6 and 7), consistent with the previous work[26]. Δ*stc*Δ*fcc*A double deletion strain showed a significant decrease in reduction activity (column 10). Therefore, evaluating the functionality of FccA^{PmC} or STC^{PmC} required performing the assay with the respective deletion mutations. Δ*stc,* FccA^{PmC} and Δ*fccA,* STC^{PmC} exhibited comparable reduction activity to WT (column 8-10). Altogether, these results show that the FP-tagged CymA, STC, and FccA are all functional in the cell. Functionality test results of other FP-tagged proteins (i.e., MtrA^{PmC}, MtrC^{PmC}, OmcA^{PmC}, Dld-II^{PmC}, Ndh^{PmC}) are shown later in Supplementary Notes 6.1

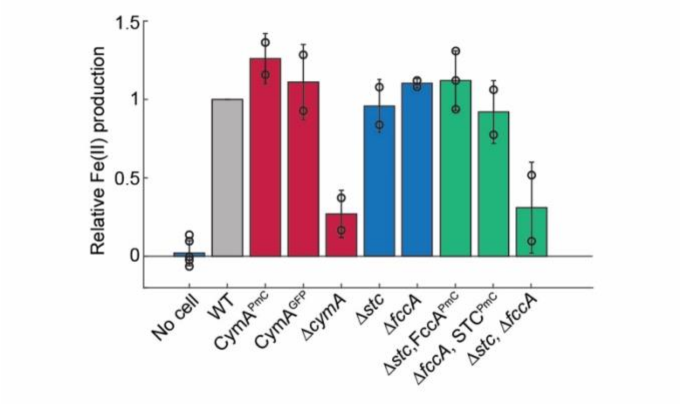

Supplementary Fig. 3 | Fe(III) reduction activities of FP-tagged proteins in the cell are comparable to wild-type. Relative amount of Fe(II) produced by FP-tagged strains (i.e., PmC- or GFP-tagged), and deletion strains grown anaerobically with lactate and ferric citrate compared with the wild-type. The Fe(II) amount was measured by Ferrozine assay (Supplementary Methods 1.4). Source data are provided as a Source Data file.

Intactness of PmC-tagged CymA, STC, and FccA. We used the additional FLAG-tag at the end of the FP tag for western blot analysis (Supplementary Methods 1.2). Western blot reveals that the tagged fusion proteins CymA^{PmC}, FccA^{PmC}, and STC^{PmC} (i.e., Protein of interest-PmC-FLAG) are predominantly intact inside the cell, regardless being grown aerobically or anaerobically (>92% intact) (Supplementary Fig. 4). The FLAG-tagged CymA^{PmC} fusion protein was observed at its expected size of 49 kDa, with merely 6-8% cleaved PmC-FLAG tag at 28 kDa (column 3 and 6). The FLAG-tagged FccA^{PmC} fusion protein was observed at its expected size of 92 kDa, with merely 5-10% cleaved PmC-FLAG (column 4 and 7). The FLAG-tagged STC^{PmC} fusion protein was observed at its expected size of 40 kDa, with <1% cleaved PmC-FLAG (column 5 and 8).

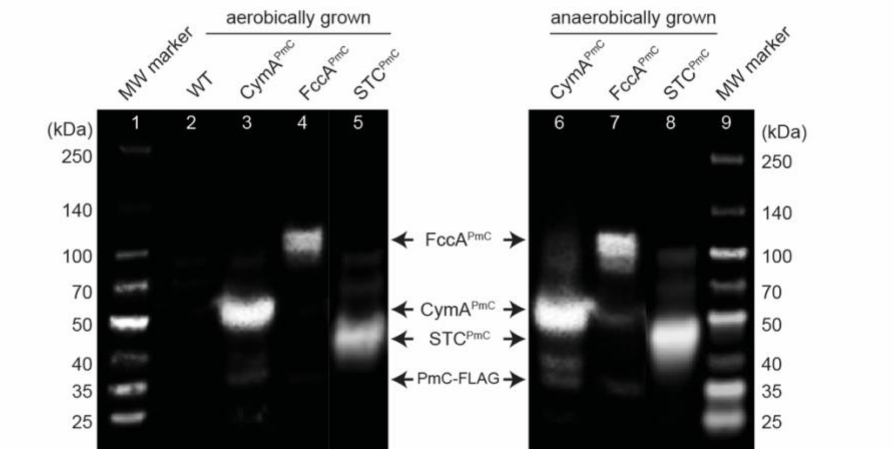

Supplementary Fig. 4 | Western blot reveals PmC-FLAG-tagged proteins are predominantly intact inside the cell. Western blot of FLAG-tagged $CymA^{PmC}$, $FccA^{PmC}$, and STC^{PmC} proteins (i.e., CymA-PmC-FLAG, FccA-PmC-FLAG and STC-PmC-FLAG) grown aerobically (left) or anaerobically (right) with lactate and ferric citrate (Supplementary Methods 1.3). Source data are provided as a Source Data file.

2. Determination of $CymA^{PmC}$ punctum size and location in the cell using machine learning segmentation

We utilized an open-source machine-learning-based (bio)image analysis tool, ilastik, to identify the $CymA^{PmC}$ puncta within the cell[28]. This process involved applying brushstrokes of various colors to different segments of the fluorescence image, with each region designated as puncta (red), cell (yellow), and background (blue) (Supplementary Fig. 5a). The software estimates the probability that each pixel in the entire image, as well as other images, belonged to each semantic classes. Subsequently, binary images representing the puncta class were generated (Supplementary Fig. 5b) and can be overlaid on the fluorescence image for visualization (Supplementary Fig. 5c). Among the cells with extracted contours (Supplementary Fig. 5d), which were then fitted with a hemisphere-capped-rod model using a custom-written MATLAB code called iQPALM[17,22] (Supplementary Fig. 5e-f; Supplementary Methods 1.5), the fraction of cells containing puncta (identified by the green contour in Supplementary Fig. 5f) was determined, and each punctum was then fitted with a circle to ascertain its location and size (i.e., diameter) (Supplementary Fig. 5f). The normalized location distribution of puncta within cells (Fig. 1e in the main text) was calculated by determining the relative punctum location within the hemisphere-capped-rod fitted cells and normalizing by the average cell size (2.42 ± 0.48 μm in length and 0.75 ± 0.12 μm in width).The punctum sizes extracted via circle fitting were proportionally consistent with diameters calculated from pixel areas, indicating that fitting with a circle represents the puncta well. The average size of the puncta of $CymA^{PmC}$ under different anaerobic growth conditions are summarized in Supplementary Table 3.

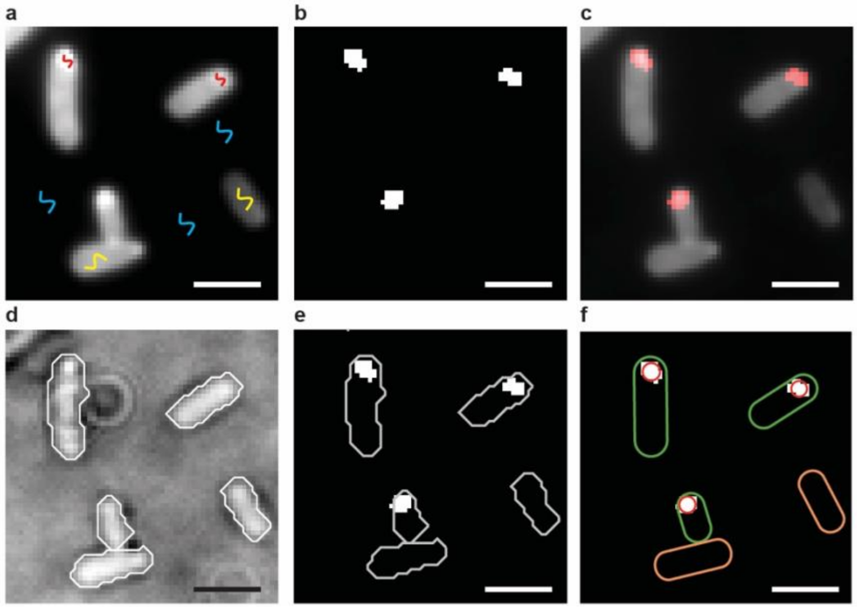

Supplementary Fig. 5 | Determination of CymA^{PmC} puncta and their sizes and locations by machine learning algorithms. **a,** Exemplary area of a fluorescence image of CymA^{PmC} cells grown anaerobically with lactate and ferric citrate. Different regions, as indicated by brushstrokes, are colored for classification: puncta (red), cell (yellow), and background (blue). **b,** Binary image from (**a**) illustrating puncta classified by the algorithm. **c,** Overlaid image of the fluorescence image and the binary image of classified puncta. **d,** Transmission image featuring cell contours extracted by the custom-written iQPALM code. **e,** Overlaid image depicting the extracted contours and the binary image of puncta. **f,** Overlaid image depicting the binary image of puncta and the contours of the cell fitted with hemisphere-cap-model and the puncta within the cells fitted with a circle. The cells containing puncta are colored in green and those without in orange. All scale bars = 1 μm.

Moreover, puncta sizes are determined from super-resolution images as well. First, we reconstructed super-resolution images as explained in Supplementary Note 13. Then, it is transformed to 2D histograms (Supplementary Fig. 6b), and the puncta are classified from the 2D histogram image by the same machine learning algorithm (Supplementary Fig. 6c). Puncta diameter obtained from such super-resolution images is 0.59 ± 0.45 μm on average (Column 2 in Supplementary Table 3), similar to the result from analyzing diffraction-limited images (0.62 ± 0.49 μm on average; Column 1 in Supplementary Table 3). To investigate whether finer structural features could be resolved within the puncta in the super-resolution images, we further reduced the super-resolution image bin size to approximately the single-molecule localization uncertainty (~40 nm. Supplementary Fig. 6d-f). However, no additional substructures were observed, which is not surprising, as possible protein spatial distribution within each condensate punctum would be beyond the resolution of super-resolution imaging. All results are summarized in Supplementary Table 3.

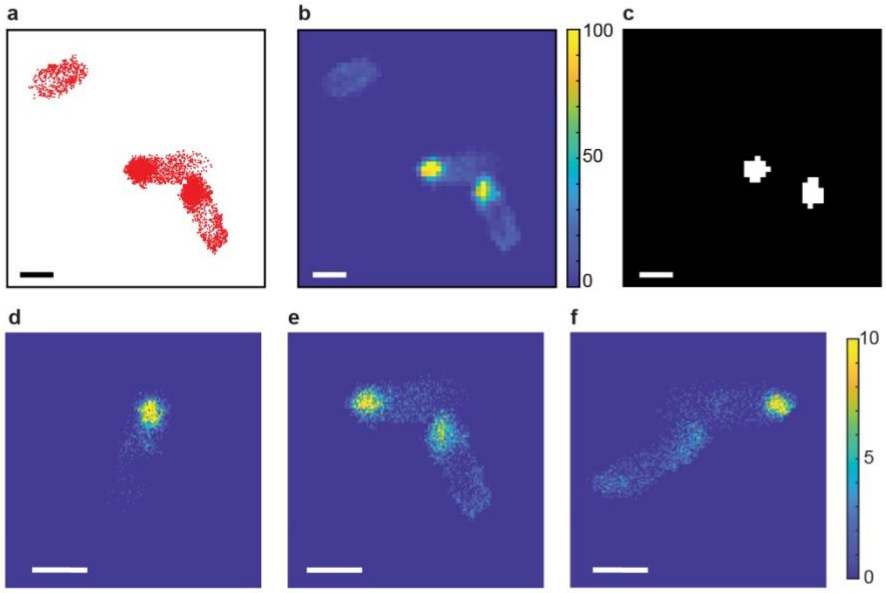

Supplementary Fig. 6 | Determination of CymA^{PmC} puncta size from reconstructed super-resolution image. **a,** Representative reconstructed super-resolution image from single molecule tracking data. Details are in Supplementary Note 12. **b,** 2D histogram image corresponding to (a). Bin size = 135 nm. **c,** Binary image from (b) illustrating puncta classified by the algorithm. **d, e, f,** Exemplary 2D-histogram image reconstructed from single molecule tracking data. Bin size = 34 nm. All scale bars = 1 µm.

Supplementary Table 3 | Size of CymA^{PmC} puncta in cells grown anaerobically with different electron donor/acceptor pairs. Diameters: mean ± s.d.

	Electron donor/acceptor pair	Puncta diameter (in µm) from pixel area – diffraction limited image	Puncta diameter (in µm) from pixel area – super resolution image	Puncta diameter (in µm) from circle fitting
1	H_2/Fumarate	0.63 ± 0.52	0.57 ± 0.45	0.44 ± 0.14
2	Lactate/Ferric citrate	0.57 ± 0.43	0.57 ± 0.46	0.42 ± 0.12
3	Fe/Fumarate	0.58 ± 0.43	0.62 ± 0.44	0.44 ± 0.16
4	Lactate/Ferric oxide	0.67 ± 0.51	0.60 ± 0.48	0.50 ± 0.18
Average		0.62 ± 0.49	0.59 ± 0.45	0.44 ± 0.16

3. Fluorescent protein tags in anaerobically grown cells can mature sufficiently by post air-exposure to render successful fluorescence imaging and there is no significant synthesis of new CymA proteins in the cell within the timescale of 4-5 h of our imaging experiment

Fluorescent proteins that are GFP variants, including PAmCherry1 (PmC) and super-folder GFP used in this study, require oxygen in the development of the fluorophore[29]. It is known that anaerobically expressed GFP can be successfully recovered to be fluorescent by post-air exposure[30]. According to the previous work[30], 60 min exposure to air is enough to achieve full recovery. In our experiment, anaerobically grown cells were exposed to air during sample preparation for imaging. It takes about an hour from collecting cells from anaerobic culture, to placing them inside the sample cell, and to completely drying the epoxy sealant of the sample cell (sample preparation section in Supplementary Methods 1.5). Thus, the fluorescent protein tag PmC/sfGFP used here should be fully matured before imaging.

It is important to highlight that the transition from anaerobic to aerobic conditions is not responsible for puncta formation because we observed the formation of CymA puncta exhibited a dependence on

anaerobic growth time (Fig. 1h). Furthermore, electrochemical imaging performed under anaerobic conditions also showed the appearance of puncta during which there was no transition from aerobic to anaerobic conditions (Fig. 2).

To verify, we compared CymA^{PmC} levels across sequentially taken images. Specifically, following ~1 h of sample preparation under aerobic conditions, we started the initial imaging session for single-molecule tracking (SMT) and single-cell quantification protein copy number (SCQPC) at one region (i.e., field of view) on the sample. This imaging process included all PmC activation and full bleaching, spanning a duration of 25 to 30 min (more details are provided in SMT and SCQPC section in Supplementary Methods 1.5). Subsequently, we transitioned to another region to repeat the same imaging protocol, encompassing activation and excitation steps. If the PmC tag had adequately matured during sample preparation, the detected average CymA^{PmC} level among the cells from region to region (later regions were taken at later times) would remain consistent. Conversely, insufficient maturation, if it were the case, would result in additional PmC maturation over the course of imaging, leading to higher cellular levels of CymA^{PmC} in subsequent images compared to the initial one.

First, as a control, we ensured that cells cultivated under aerobic conditions demonstrated stable CymA^{PmC} levels throughout the imaging process, consistent with expectations (Supplementary Fig. 7a). Then, we validated that cellular concentrations of CymA^{PmC} cultured anaerobically (specifically, with lactate and ferric citrate as electron donor and acceptor, as per the dataset) exhibited consistent concentration levels through the images taken sequentially, indicating that maturation was completed during sample preparation before the initial image was taken (Supplementary Fig. 7b). Hence, sufficient time was allotted for tag maturation prior to the first image acquisition.

Additionally, it is worth noting that we imaged a total of 6-8 different regions, requiring approximately 4-5 h from sample preparation to completion of imaging for each sample. Importantly, we observed no discernible elevation in CymA concentration during this timeframe, suggesting that no significant synthesis of new CymA proteins within this temporal range.

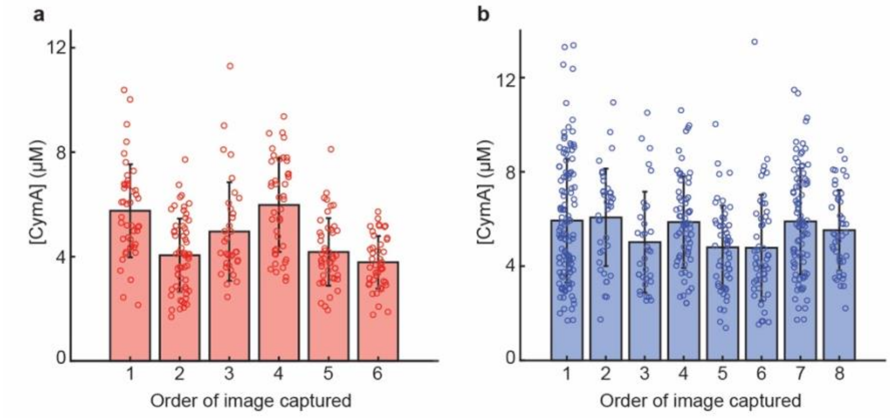

Supplementary Fig. 7 | Anaerobically expressed fluorescent protein tags matured sufficiently during sample preparation by post air-exposure. Representative data of average cellular CymA^{PmC} concentration across different images captured at 25-30 min intervals; each image is on a different region. **a,b,** Both aerobically (**a,** sample size n = 45, 59, 35, 42, 45, 43) and anaerobically (**b,** sample size n = 116, 38, 33, 62, 53, 50, 85, 43) cultured cells maintain consistent concentration levels across the sequential acquisition of images. The post-maturation of PmC, a process requiring oxygen for fluorescence, is effectively completed prior to the first image acquisition. Source data are provided as a Source Data file.

4. Determination of lag time and transition time of CymA punctum formation in the cell by fitting with a sigmoidal function

Our results show that the formation of CymA punctum over time followed a sigmoidal time profile both at the ensemble level and at the single-cell level (e.g., Fig. 1h and Fig. 2e in the main text). To quantify this temporal behavior of punctum formation, we employed a sigmoidal function with two asymptotes at both ends (Supplementary Fig. 8). This function, defined by four parameters (Equation S1), can satisfactory

describe the temporal behaviors of CymA punctum formation (see fittings in Fig. 1h and Fig. 2e in the main text): a denotes the lower asymptote; b denotes difference between the upper and lower asymptotes; c denotes the inflection point along the time axis; d is an exponential time constant, determining the steepness of the sigmoidal rise part of the curve.

$$f(t) = a + \frac{b}{1 + \exp\left(-\frac{t-c}{d}\right)} \qquad \text{Equation S1}$$

We defined the transition time as $4d$, i.e., four times of the exponential time constant (i.e., covering a time-width $2d$ on both sides of the inflection point c) and defined the lag time as the interval from $t = 0$ to the onset of the transition time (Supplementary Fig. 8). All observations related to CymA punctum formation, including the fractional population of the cells showing puncta (Fig. 1h), skewness of fluorescence pixel intensity distribution (Fig. 2e), and menaquinone production (Fig. 3h) vs. time trajectories, were subjected to sigmoidal function fitting and the lag times and transition times were determined in the same way.

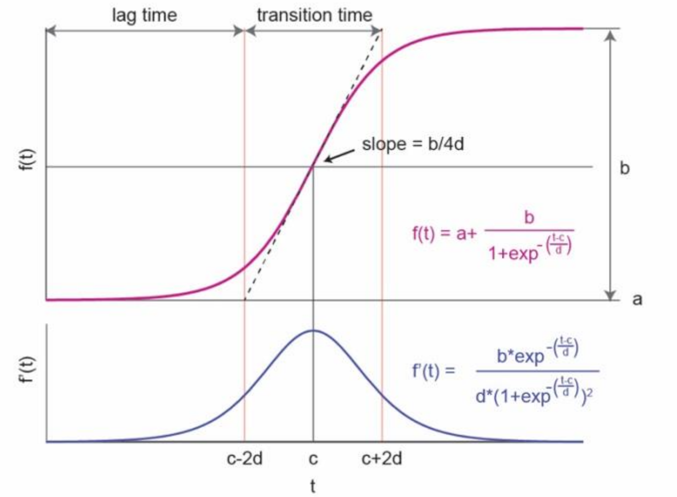

Supplementary Fig. 8 | Graph of sigmoidal function (top) and its first derivative (bottom) with denotation of four parameters. a denotes the lower asymptote; b denotes difference between the upper and lower asymptotes; c denotes the inflection point; d is the exponential time constant, determining the steepness of the rise part of the curve. We defined the transition time as $4d$, four times of the exponential time constant, straddling the inflection point, and the lag time as the interval from $t = 0$ to the onset of the transition time.

5. Skewness provides quantitative analysis of temporal dynamics of punctum formation in single cells

To quantify the temporal dynamics of punctum formation in single cells, we used the skewness of fluorescence pixel intensity distribution among all the image pixels of each cell. Skewness is the degree of asymmetry observed in a distribution. When CymA^{PmC} is homogeneously distributed on the cell envelope, the fluorescence intensity of individual pixels in a cell in a wide-field 2-D projection image would have a broad distribution without any notable tail (Supplementary Fig. 9a and b, 0 – 2.5 h). This broad distribution comes from a couple of factors: 1) slightly higher intensity on the cell envelope than inside the cell; 2) slight mismatch between the extracted cell contours (which is slightly larger) and actual cell morphology due to the image's pixelation and diffraction-limited spatial resolution. The pixels at the contour edge of the cell have low intensity. Even with these factors, the pixel intensity distribution is fairly symmetric and gives skewness value close to zero. With increasing observation time and time-lapse imaging, the accumulated laser exposure results in gradual fluorescence bleaching, as reflected by an overall shift of the fluorescence pixel intensity distribution to the lower values (Supplementary Fig. 9a and b). However, such

fluorescence bleaching occurs spatially equally across all pixels, so the symmetry (or asymmetry) of the pixel intensity distribution is not affected by this photobleaching significantly. When $CymA^{PmC}$ forms a punctum, pixels in a punctum have higher intensity while others outside of the punctum are comparably much lower (e.g., Supplementary Fig. 9a and b; after 3 h). Correspondingly, a distribution tail emerges in the pixel intensity distribution on the right, higher intensity side (Supplementary Fig. 9a and b, emerged at 3 h and stayed up to 6 h, as highlighted in green shade), giving positive skewness. As more $CymA^{PmC}$ gather in a punctum, the intensities of the pixels in the punctum increase, while those outside the punctum decrease due to lower molecule amounts and show lower intensity. This led to a further increase in skewness. Thus, skewness over time effectively describes the temporal emergence of puncta in a single cell (Supplementary Fig. 9c). By fitting skewness-vs-time profile with a sigmoidal function, we can extract the lag time and the transition time regarding punctum formation systemically (Supplementary Fig. 9c). Additionally, this skewness analysis is consistent with the result from the machine learning (ML) algorithm; cells with a punctum, identified by ML algorithm, exhibit higher skewness Supplementary Fig. 9d, orange), while cells without a punctum show lower skewness, with the mean value near zero (Supplementary Fig. 9d, grey).

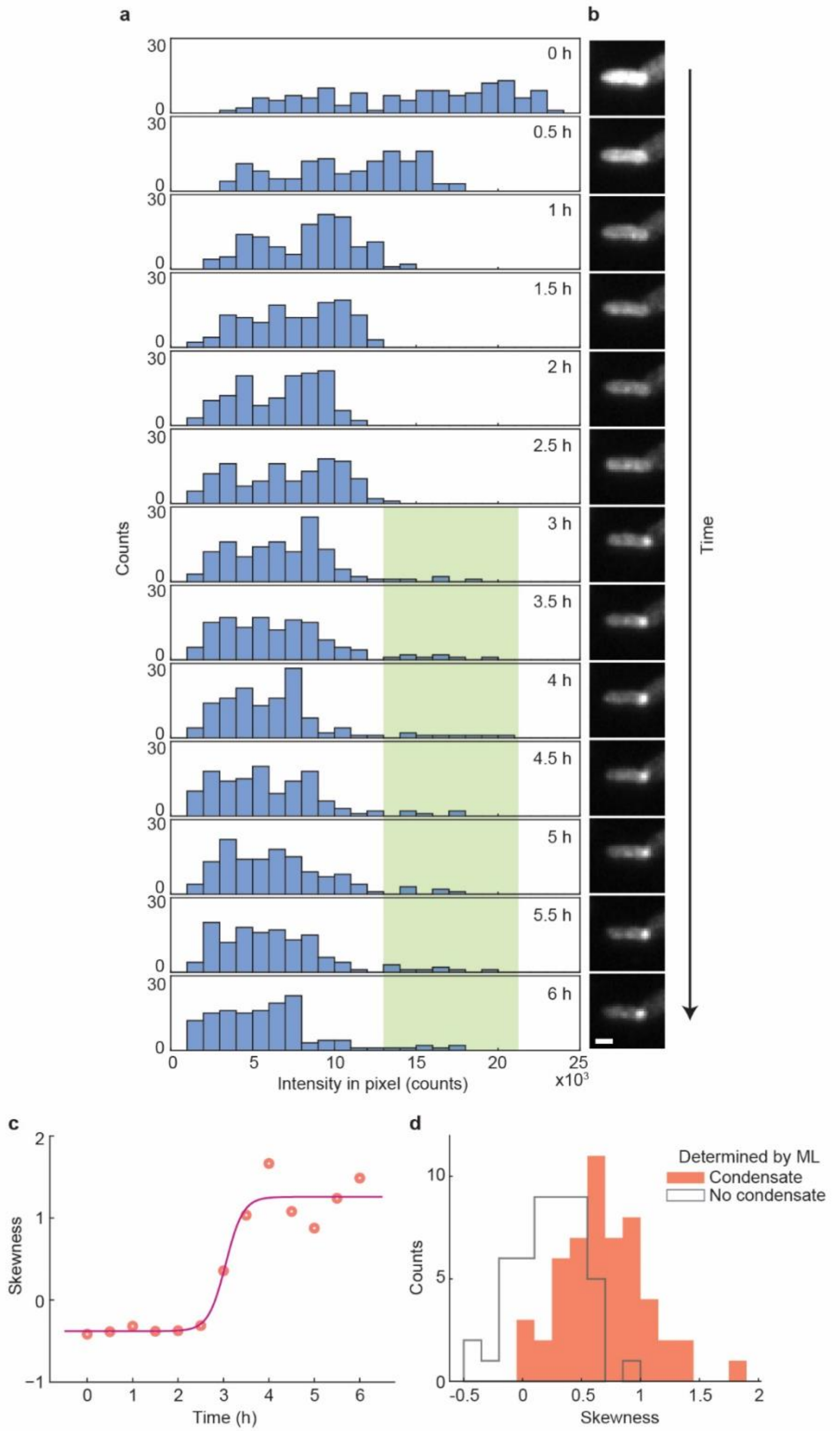

Supplementary Fig. 9 | Skewness calculation from wode-field single-cell fluorescence images. a, Intensity distributions of pixels in a cell, and **b,** the corresponding cell fluorescence images as a function of time. The cells were placed on ITO glass and the potential −0.48 V (vs. Ag/AgCl) was applied. Emergence of punctum in the cell causes emergence of a tail on the high intensity side of the distribution (green shade). The experimental details are in Supplementary Methods 1.5. Scale bar: 1 μm. **c,** Skewness vs time (open circle) corresponding to (**a** and **b**) fitted with a sigmoidal function (pink line). **d,** Histograms of skewness of individual cells grown anaerobic conditions (lactate/ferric citrate) for 2 days and the punctate in the same cells were also determined by

machine learning algorithms. Cells with a punctum (orange filled, n = 53) showed positive skewness while cells without a punctum (gray line, n = 48) showed skewness near zero, as expected. Source data are provided as a Source Data file.

6. No colocalization between CymA and outer- or inner-membrane EET proteins under anaerobic growth conditions with active EET

6.1 Tagging PmC to outer and inner membrane EET proteins (OmcA/MtrCAB, Dld-II, Ndh) and their spatial distributions in the cell do not show punctate features under anaerobic growth

To probe whether the outer or inner membrane EET proteins may colocalize with CymA punctum, we genetically fused PmC tags to the C-termini of OmcA, MtrC/A/B, Dld-II, and Ndh, one at a time at their respective chromosomal loci (Fig. 1a in the main text, Supplementary Methods 1.2). To ensure that the tagging process did not interfere with the EET function of these proteins, iron reduction assays were conducted using ferrozine to compare the WT cells with the tagged strain and the corresponding deletions (Supplementary Methods 1.4); the results confirmed the preservation of the functionality for the PmC-tagged OmcA, Did-II, and Ndh (Supplementary Fig. 10a). Note that given that MtrC serves as another metal reductase alongside OmcA in the outer membrane, the deletion of the *omcA* gene did not completely impair metal reduction capability, but still showed less metal reduction ability. Unfortunately, the PmC-tagged MtrC/A/B are nonfunctional (Supplementary Fig. 10b), preventing the use of FP-tagging to visualize their spatial distribution in the cell. However, it has been previously reported that OmcA and MtrCAB form a complex[31]. By introducing the PmC tag to OmcA, we could visualize the spatial distribution of the OmcA/MtrCAB outer-membrane protein complex together.

To visualize the spatial distribution, we first cultured each strain under anaerobic condition with lactate/ferric citrate for OmcA and Dld-II, and NADH/ferric citrate for Ndh, where all three proteins (OmcA, Dld-II, and Ndh) should participate in the outflow EET with CymA in anaerobic respiration (Fig. 1a in the main text). However, no protein puncta were observed in the cell; instead, all three proteins exhibited homogeneous distribution across the cell envelope (Supplementary Fig. 10c-e), indicating that these outer and inner membrane EET proteins do not spatially colocalize with CymA punctum during EET. In the case of OmcA, these findings align with previous reports indicating the absence of any specific pattern in the localization of QD-tagged OmcA and MtrC along the cell surface[32], as well as similar observations made through electron cryotomography[33].

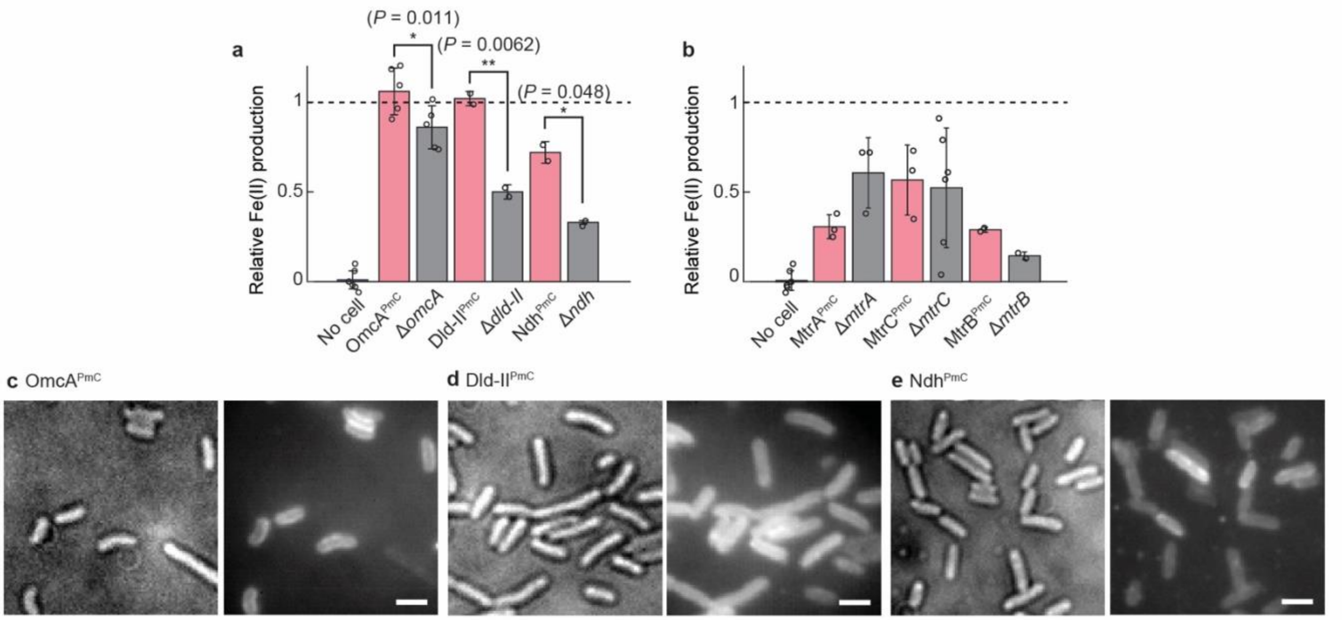

Supplementary Fig. 10 | Outer and inner membrane EET proteins do not show punctate patterns during EET. a,b, Relative Fe(II) production amount compared with WT measured by ferrozine reduction assay (Supplementary Methods 1.4). Mean (bar) and s.d. (error bars) are calculated from repeated experiments (a, sample size n = 7, 5, 5, 2, 2, 2, 2; b, sample size n = 7, 3, 3, 3, 5, 2, 2). P-value is calculated by two-sided t-test ($P < 0.05$, significant). **c-e,** Bright-field transmission images (left), and wide-field epi-fluorescence images (right) of ensemble-photoactivated (c) OmcA^{PmC}, (d) Dld-II^{PmC}, and (e) Ndh^{PmC}, cultured under anaerobic conditions (lactate/ferric citrate). Proteins are homogenously distributred across the cell envelope. Scale bars = 2 µm. Source data are provided as a Source Data file.

6.2 No significant colocalization of CymA puncta with outer membrane extensions (OME) generation spots

Previous research has revealed that multiheme cytochromes MtrC and OmcA in *S. oneidensis* can localize along the OME and shuttle respiratory electrons to electron acceptors located micrometers away from the cell[34]. We examined whether the locations of CymA puncta could possibly be associated with OME generation spots on the outer membrane.

Here we performed 2-color imaging of CymA^{PmC} and the bacterial membrane stained with a fluorescent dye. First, we grew CymA^{PmC} cells anaerobically with lactate/ferric citrate then imaged ensemble-photoactivated CymA^{PmC} in the red-channel (Supplementary Fig. 11a and d; Supplementary Methods 1.5). Subsequently, the cells were stained with SynaptoGreen C4 (Biotium), a lipophilic green fluorescent dye that inserted into the cell membrane and was previously shown to highlight OMEs in *S. oneidensis* cells[34], and we imaged OMEs in the green-channel (Supplementary Fig. 11b, c, e, f).

A recent study reported that contacting with a surface triggers OME production in *S. oneidensis* cells[35]. Indeed, 3 h after placing cells and staining on the glass slide, OMEs became observable, which was not the case 1 hour after placing the cell and staining (Supplementary Fig. 11b-vs.-c, e-vs.-f; more noticeable in zoom-in images, Supplementary Fig. 11-(i-v)). We analyzed 43 OME generation spots on many cells, assessing their colocalization with CymA^{PmC} puncta. We first examined cells with observable OMEs, acknowledging that OMEs might not be observable if they are not long enough to be seen at diffraction-limited resolution, as such OMEs were previously reported to be ~100 nm in width[33]. Some cells exhibited colocalization of a punctum and a OME generation point (Supplementary Fig. 11-(ii and iii)); however, other cases were also easily observed: (1) OMEs and CymA puncta were at different locations (Supplementary Fig. 11-(iv)); (2) OMEs were observed in cells without a punctum (Supplementary Fig. 11-(v)). We also investigated cell with a punctum and found multiple cells did not have observable OMEs (Supplementary Fig. 11-(vi)).

Our findings revealed that among cells with OMEs, 41% of these OME generation spots lacked a punctum, 13% exhibited a punctum at different locations, and 46% showed colocalization. Additionally, there were multiple cells with a punctum did not have observable OMEs. Altogether, these results suggest no significant association between OME generation and CymA puncta. This conclusion is also consistent with the fact that after anaerobic growth in solution and before the cells had sufficient time contacting the slide for OME formation, CymA puncta were already formed (Supplementary Fig. 11a, d).

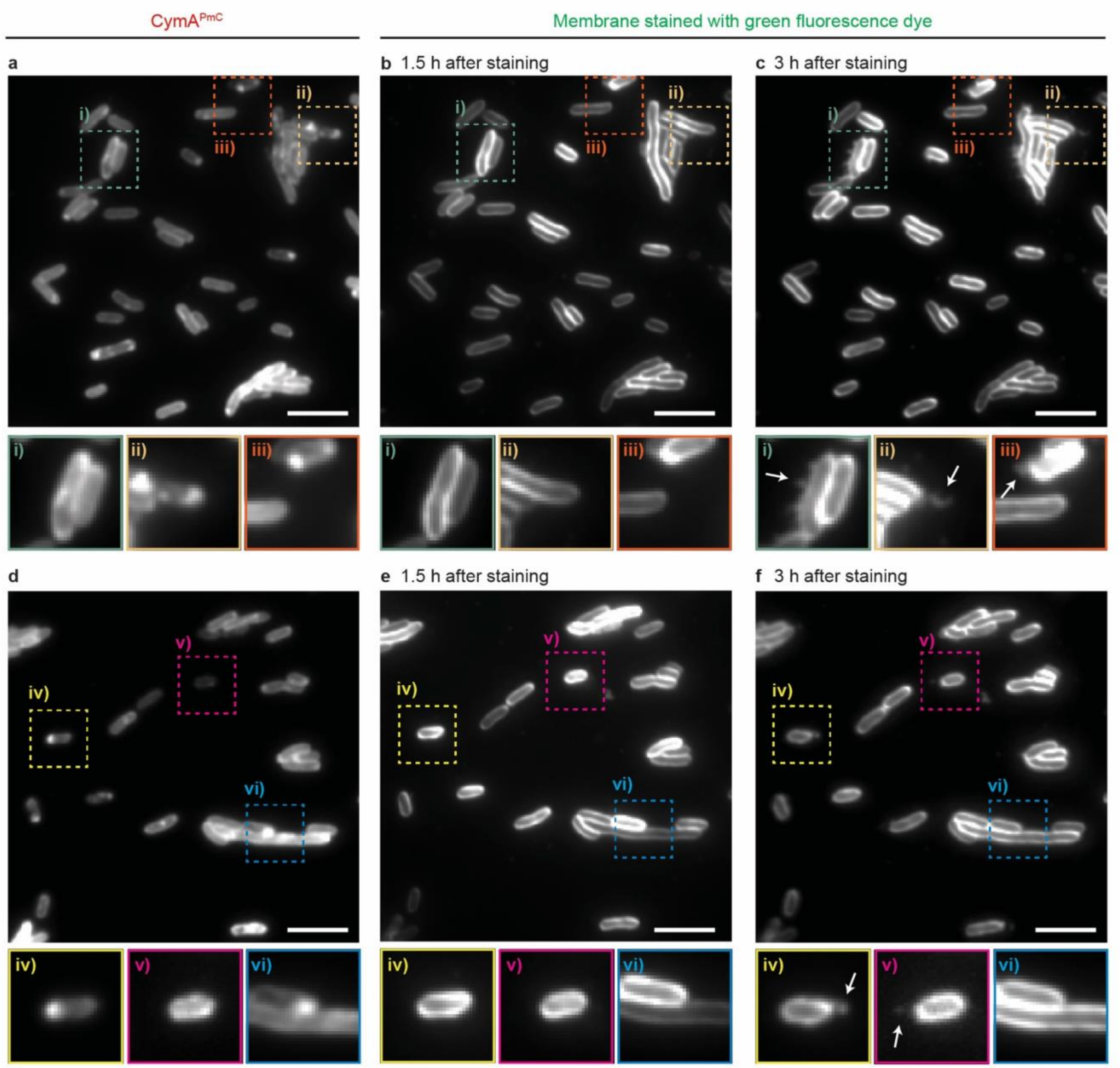

Supplementary Fig. 11 | No significant colocalization between CymA puncta and outer membrane extensions (OMEs). a-c, Wide-field epi-fluorescence images of ensemble-photoactivated anaerobically grown (lactate/ferric citrate) CymA^{PmC} strain (**a**, red channel), where its membrane was stained with SynaptoGreen (**b,** 1.5 hour after staining, **c,** 3 hour after staining, green channel). **d-f,** Same as a-c, but another region in the same experiment batch. Bottoms: zoom-in of the dashed box in a-f. White arrows in zoom-in images denote the generated OME. Scale bars = 5 μm.

7. Challenges in measuring photoanodic current for electron outflow

We measure the photocathodic current of individual *S. oneidensis* cells to access their electron uptake capability in EET (Fig. 4 in the main text). It has been previously reported that *S. oneidensis* can also transport electrons directly to the electrode in the outflow direction. Thus, measuring photoanodic current could be an alternative method to investigate the enhanced EET ability of CymA puncta. However, the valence bands of many semiconductors are relatively deep (e.g., Cu_2WS_4: ~1.5 V vs. Ag/AgCl[25], $BiVO_4$: ~2.3 V vs. Ag/AgCl[36], CdS: ~1.6 V vs. Ag/AgCl[37], TiO_2 ~2.5 vs. Ag/AgCl), making the photogenerated valence band holes in these semiconductors powerful oxidants, capable of even oxidizing water molecules (which our lab also studied in separate research projects[38,39]). Such photoanodic water oxidation also generate reactive oxygen species (e.g., hydroxyl radicals), causing significant damage to cellular components including lipids, proteins, and DNA, which can be fatal to cell viability[40].

Electron transport chain (ETC) relies on a flow of electrons from reducing equivalents (e.g., NADH, $FADH_2$, or Quinones) through a series of protein complexes to a terminal electron acceptor. With photo-induced holes from semiconductors with a low valence band, these reducing equivalents are prone to oxidation. Ubiquinone (–0.09 V vs. Ag/AgCl), menaquinone (–0.27 V vs. Ag/AgCl), flavin mononucleotide (FMN, –0.39 V vs. Ag/AgCl), flavin adenine dinucleotide (FAD, -0.42 V vs. Ag/AgCl), and nicotinamide adenine dinucleotide (NAD^+, –0.52 V vs. Ag/AgCl) are all susceptible to this oxidation process[41,42]. Therefore, photoanodic measurements are complicated by these oxidative processes, and we opted to measure photocathodic current to assess the electron uptake capability in EET.

8. Reactive oxygen species (ROS) does not play a significant role in enhancing electron-uptake capabilities by cells in our study

In photoelectrochemical measurement, we used Cu_2WS_4 to generate excited electrons locally using 405 nm light irradiation. While Cu_2WS_4 film can also generate reactive oxygen species (ROS) with visible light irradiation, possibly contributing photocurrent measurement[43], ROS generation should be minimal in our study under our photocathodic current measurements (i.e., reductive currents where photogenerated electrons in Cu_2WS_4 are donated out while the photogenerated holes are extracted away by the back-contact ITO electrode):

1) The photocurrent measurements were conducted under anaerobic conditions, eliminating molecular oxygen as a potential electron acceptor and thereby suppressing ROS formation via reduction pathways.
2) The efficient extraction of photogenerated holes through the ITO back-contact electrode was imposed by applying a sufficiently negative electrochemical potential, which prevents water oxidation and associated oxidative pathways for ROS generation. The experimental configurations are illustrated in Supplementary Fig. 1c.

Moreover, even if ROS generation or reductants (e.g., fumarate) contribute to photocurrent generation partly, it would be the same for off-cell regions or for on-cell without puncta. Therefore, the enhanced photocathodic current observed specifically in cells exhibiting CymA puncta cannot be attributed to general ROS reactivity or reactant availability.

Importantly, we observed no significant changes in electrochemical or physiological properties of the cell, which would occur if there were significant oxidative stress during the measurements: (i) the photocurrent enhancement remained stable over multiple cycles (10 s off, 15 s on, total 75 s) of light off/on illumination, and (ii) post-measurement imaging revealed no morphological changes, which would happen with cell lysis or death.

As Dai *et al* have reported that the interface of biomolecular condensates can drive spontaneous ROS generation in living cells[44], we measured ROS level in *S. oneidensis* pre-grown anaerobic with lactate/ferric citrate (Supplementary Fig. 12). In our experiments, however, we did not observe significant ROS generation within the biomolecular condensates (white arrows in Supplementary Fig. 12, b-vs.-c and e-vs.-f). Instead, higher H_2O_2 levels were observed in inclusion bodies (insoluble aggregates) visible in bright-field transmission images (yellow arrows in Supplementary Fig. 12a and c). The observation of higher H_2O_2 in inclusion bodies agree with the previous work that interfaces between substantial phase

differences may promote H_2O_2 generation[44]. The fact that higher H_2O_2 was not observed in CymA condensate might stem from differences in the physical properties (i.e., liquidity) of the biomolecular condensates, which are protein-dependent. Therefore, CymA biomolecular condensates itself did not appear to generate significant ROS that could contribute to the measured photocurrent.

While ROS produced by biomolecular condensates may contribute to the photocurrent, this effect is likely negligible, because:

1) Previous studies showed that ROS, particularly H_2O_2, remained confined within the condensates in both in vitro and in vivo system[44,45]. In the case of CymA in *Shewanella*, which is localized to the inner membrane, the spatial separation between the inner and outer membranes (~23.5 nm; measured by electron microscopy[46]) makes it unlikely that ROS generated within the inner membrane-associated condensates would efficiently diffuse across membranes to reach the Cu_2WS_4 film electrode to significantly impact the photocurrent.
2) A previous study showed that through direct electrochemical measurements, ROS encapsulated inside stress granules contributed to just a few tens of pA[45]. Although it is hard to directly compare with our experiments due to different biological systems, their reported current magnitude is an order of magnitude smaller than our measurement, –0.40 ± 0.07 (s.e.m.) nA.

All the above evidence collectively led us to conclude that ROS generation in our system is likely insignificant and does not play a significant role in enhancing electron-uptake capabilities by cells.

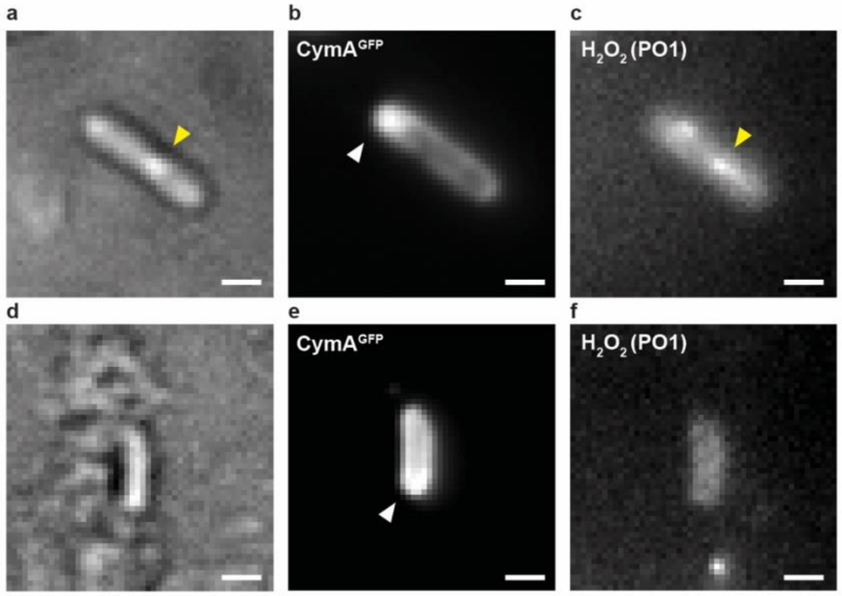

Supplementary Fig. 12 | Reactive oxygen species level measurement in live *S. oneidensis*. Peroxy orange 1 (PO1) assay for the investigation of spatial distribution of H_2O_2. **a-c,** Bright-field transmission (a) and wide-field epi-fluorescence images of ensemble-photoactivated anaerobically grown (lactate/ferric citrate) $CymA^{GFP}$ strain (b, green channel), where generated H_2O_2 was assayed with PO1 (c, red channel). **d-f,** Same as a-c, but another region in the same experiment batch. Yellow arrows denote the inclusion body in the cells, identifiable in the bright-field transmission image (a). White arrows denote the CymA puncta, visible in $CymA^{GFP}$ fluorescence images (b, e). Scale bars = 1 nm.

9. Discussion about single-cell current measurement

There are previous studies that have quantified the electrochemical currents in the outflow direction produced by single electroactive bacteria. Liu et al.[47] reported that individual *Shewanella loihica* (PV-4) cells generated 200 – 400 fA outflow current (i.e., anodic current), when measured on an ITO microelectrode under a constant potential of +200 mV vs. Ag/AgCl. Similarly, McLean et al.[48] deduced that single *Shewanella oneidensis* (MR-1) cells produced ~200 fA outflow current on graphite with a constant potential of +230 mV vs. Ag/AgCl; This single-cell current was derived from the measured current

of multiple bacteria divided by the number of cells. Additionally, Gross et al.[49] reported that *S. oneidensis* on an ITO electrode produced 15 – 100 fA outflow current at a constant potential of +400 mV vs. Ag/AgCl, noting significant heterogeneity among individual cells in current production. Besides microelectrode-based methods, Tang et al.[50] measured single *Shewanella oneidensis* (MR-1) cell current with a magnetite nanoprobe, reporting that the cells produced the outflow current of 0-2.7 fA.

Beyond *Shewanella* species, Jiang et al.[51] investigated the single-cell current of *Geobacter sulfurreducens* (DL-1). They found that individual *G. sulfurreducens* cells produced ~100 fA outflow current on a fabricated transparent Ti/Au finger electrode at an applied potential of +400 mV vs. Ag/AgCl. Collectively, these studies support that single electroactive bacterium can generate outflow, anodic currents in the range of a few hundred femtoamperes under a constant anodic potential.

In contrast, our photoelectrochemical current measurement (Δi, Fig. 4 in the main text) demonstrated that single *S. oneidensis* cells can uptake ~400 pA (i.e., cathodic current), which is three orders of magnitude higher than previously reported currents. We propose several possible reasons for our measured much larger current magnitude: (1) Direction of EET is different: Previous studies measured EET in the outward direction, whereas we quantified electron uptake by cells. The amount of electron flux may naturally vary depending on the flow direction. (2) When a cell is engaged in outflow EET, some of the outflowing electrons might go to other cellular components for metabolism and biosynthesis and thus not collected by the electrode, and as such the electrochemical current would be an underestimate of the overall EET outflow. Conversely, in our measuring electron uptake, the measured current going through the photoelectrode account for the maximal flux of electrons assimilated by the cells. (3) In our measurement, the electron uptake is driven by the photo-generated conduction band electrons of the Cu_2WS_4 semiconductor; the applied potential on the back contact electrode ITO serves to extract the photogenerated holes in Cu_2WS_4. The high photogenerated electron flux, driven by the focused laser excitation, may lead to a significantly higher single-cell current than what was previously measured through an electrode (instead of a photoelectrode as we report here).

Furthermore, Jiang et al. and Gross et al. reported heterogeneity in current production among cells. For *G. sulfurreducens*, only 30% of observed cells exhibited measurable current, and 4 out of 27 cells were actively respiring at the electrode for *S. oneidensis*. The origin of this heterogeneity in their studies remains unclear, but our findings suggest it may be attributable to the heterogeneous formation of cellular puncta.

10. Discussion about CymA expression levels and EET function

While previous studies have reported that overexpression of CymA can enhance extracellular electron transfer (EET) activity[52], our study does not involve CymA overexpression. It is worth noting that all our observations were made under physiological expression levels. We demonstrated that cellular CymA concentrations, either with or without puncta in cells, showed no significant correlation with their photocurrent Δi in Fig. 4d in the main text. In addition, we also investigated the correlation between CymA concentration and photocurrent density (Supplementary Fig. 13). Here the photocurrent density was calculated by dividing the single-cell current by the area of the cell in contact with the Cu_2WS_4 thin film within the laser-illuminated region (Fig. 4a). Consistent with the result in Fig. 4, cells with puncta showed significantly more negative current density compared to cells without puncta (Supplementary Fig. 13, right panel), even though their cellular concentration of CymA was similar (top panel), indicating again that CymA expression level alone is insufficient to drive EET. Instead, our data indicate that the formation of CymA puncta is a critical determinant of EET functionality. Interestingly, cells with puncta showed a marginal negative correlation, with Pearson's cross correlation coefficient of -0.28 with a P-value of 0.042) (Supplementary Fig. 13, solid red symbols), suggesting that more CymA in the puncta could potentially facilitate slightly better EET *at the per-contact-surface-area level*, which would be consistent with previous finding that cells overexpressing CymA show higher EET activities[52]. However, it's important to note that our study does not involve CymA overexpression—all observations here were made under physiological expression levels.

While it is plausible that non-physiological overexpression could influence both spatial organization and EET performance, potentially by altering protein localization dynamics or stoichiometry, such scenarios are beyond the scope of this study. Future work could explore how overexpression level perturbations affect spatial compartmentalization and EET efficiency.

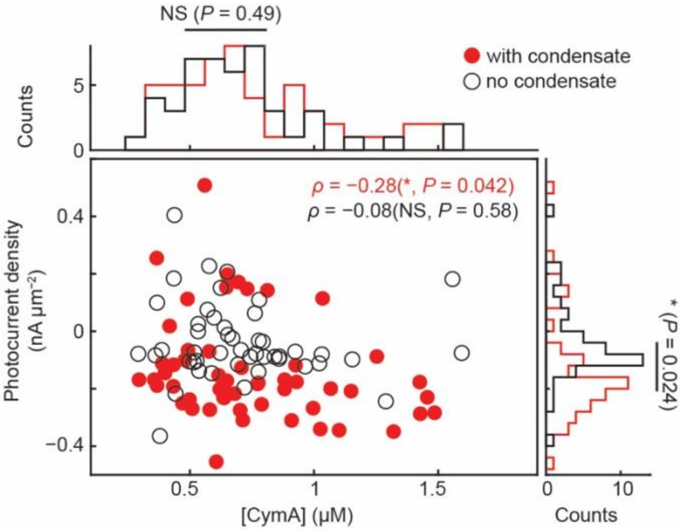

Supplementary Fig. 13 | Marginal negative correlation between cellular concentration of CymA and photocurrent density of single cells. Photocurrent density calculated by dividing the single-cell current by the area of the cell occupied on the Cu_2WS_4 thin-film within the laser illumination spot (Fig. 4). Circles: individual cells containing puncta (solid red) or no puncta (open black). Denoted are Pearson's correlation coefficient ρ for cells with puncta (red) and cells without (black) and their P values (two-sided t-test: P < 0.05, significant). Top/right: Projected histograms. Statistical significance was determined using Welch's two-sample t-test (NS, not significant; P < 0.05, significant). Source data are provided as a Source Data file.

11. No significant difference in cellular concentrations of STC/FccA/OmcA for anaerobically grown cells that had formed puncta vs. those that had not

We investigated the cellular concentrations of other EET partner proteins (i.e., periplasmic proteins, STC and FccA, and outer membrane protein, OmcA; Fig. 1a) in cells that had formed puncta and that had not to probe whether the anaerobic upregulation of these partner proteins[53] (Supplementary Fig. 28) contributes to enhanced EET. Initially, we constructed double-tagged strains containing PmC-tagged protein of interest and sfGFP-tagged CymA (i.e., STC^{PmC} + $CymA^{GFP}$, $FccA^{PmC}$ + $CymA^{GFP}$, and $OmcA^{PmC}$ + $CymA^{GFP}$) (Supplementary Methods 1.2), and each strain was grown anaerobically (lactate/ferric citrate). Subsequently, cells were imaged in the red channel (PmC) to quantify protein concentration and in the green channel ($CymA^{GFP}$) to determine the presence of CymA puncta (Supplementary Methods 1.5). This approach allowed us to compare the concentration of the protein of interest between cells with and without CymA puncta, identified using machine learning algorithms (Supplementary Notes 2).

Our analysis revealed no significant differences in the concentration of STC, FccA, and OmcA in cells that had vs. those that had not formed CymA punctum (Supplementary Fig. 14). While a slight difference was observed for STC, with a higher mean concentration in cells that had not formed puncta by ~8% (Supplementary Fig. 14a), this difference is opposite to that cells with CymA puncta have higher photocathodic current and thus cannot be the reason for the ~100% enhancement of photocathodic current (Fig. 4d in the main text). These findings suggest that these EET partner proteins are adequately upregulated under anaerobic growth conditions to reach sufficient protein levels in the cell regardless CymA punctum formation. This observation also aligns with the known regulation of anaerobic respiration in *S. oneidensis* through the same cyclic AMP receptor protein (CRP)[54,55], which leads to the transcriptional activation of proteins such as STC, FccA, and OmcA upon exposure to anaerobic conditions.

In conclusion, the results that there are no significant differences in the concentration of periplasmic or outer membrane proteins in cells that had formed CymA puncta vs. those that had not reinforce the significance of CymA punctum formation as the dominant factor in rendering EET in cells.

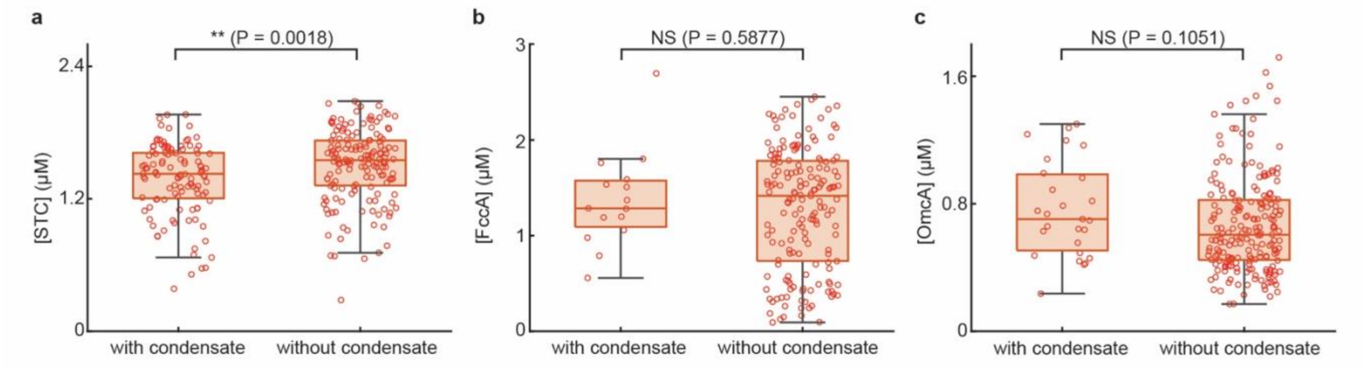

Supplementary Fig. 14 | No significant concentration difference in outer- or periplasmic proteins in cells that had vs. that had not formed puncta grown anaerobically. a-c, Protein (**a,** STC; **b,** FccA; **c,** OmcA) concentration comparison in cells with and without CymA punctum, where cells were grown anaerobically (lactate/ferric citrate). Sample number *n* = 112, 159; *n* = 15, 171; *n* = 27, 204. Box plots show medians with 1.5 IQR whiskers. Source data are provided as a Source Data file.

12. Analysis of diffusion dynamics of CymA in the cell to resolve its minimal number of diffusion states as well as the associated diffusion constants (*D*) and fractional populations (*A*)

12.1 Inverse transformation of confined displacement distribution (ITCDD) algorithm to deconvolute the cell confinement effect on the displacement length distribution obtained from single-molecule tracking

Single-molecule tracking gives the position vs. time trajectories of each tracked protein, which encodes the protein's mobility information. To analyze the mobility dynamics of a single protein, the first displacement length *r* of each tracking trajectory was collected and compiled to build a displacement length distribution (Supplementary Fig. 15a and d). However, this displacement distribution is distorted due to the finite size of the bacteria cell and therefore the confinement of the protein diffusion, as well as the projection of single molecule locations from 3D space to a 2D plane since the *z*-position was not determined during our imaging. This distortion causes an inaccuracy in determining the minimal number of actual diffusion states, their diffusion constants, and the states' fractional populations, and it can sometimes even lead to some artificial diffusion states. We therefore used an inverse transformation of confined displacement distribution (ITCDD) method to deconvolute out the confinement effect that were previously developed and shown to be effective for both cytosolic and membrane proteins in bateria[56,57]. The original probability density function (PDF) of the confined displacement distribution PDF_{CS} was multiplied by the inverse of the confinement transformation matrix ($[CTM]^{-1}$), which defines the relation, for any given displacement length on the cell membrane (or in the cytosol), of the resulting distorted displacement length distribution after projection, for a cell of a known geometry, which is determined from the cell's transmission image. The PDF(*r*) in free space (PDF_{FS}) can then be obtained through Equation S2 (Supplementary Fig. 15b and e). As CymA is an inner-membrane protein (Main Text Fig. 1a), the confinement transformation matrix ($[CTM]^{-1}$) corresponding to membrane proteins was used.

$$\mathrm{PDF_{FS}} = [\mathrm{CTM}]^{-1} \cdot \mathrm{PDF_{CS}} \qquad \text{Equation S2}$$

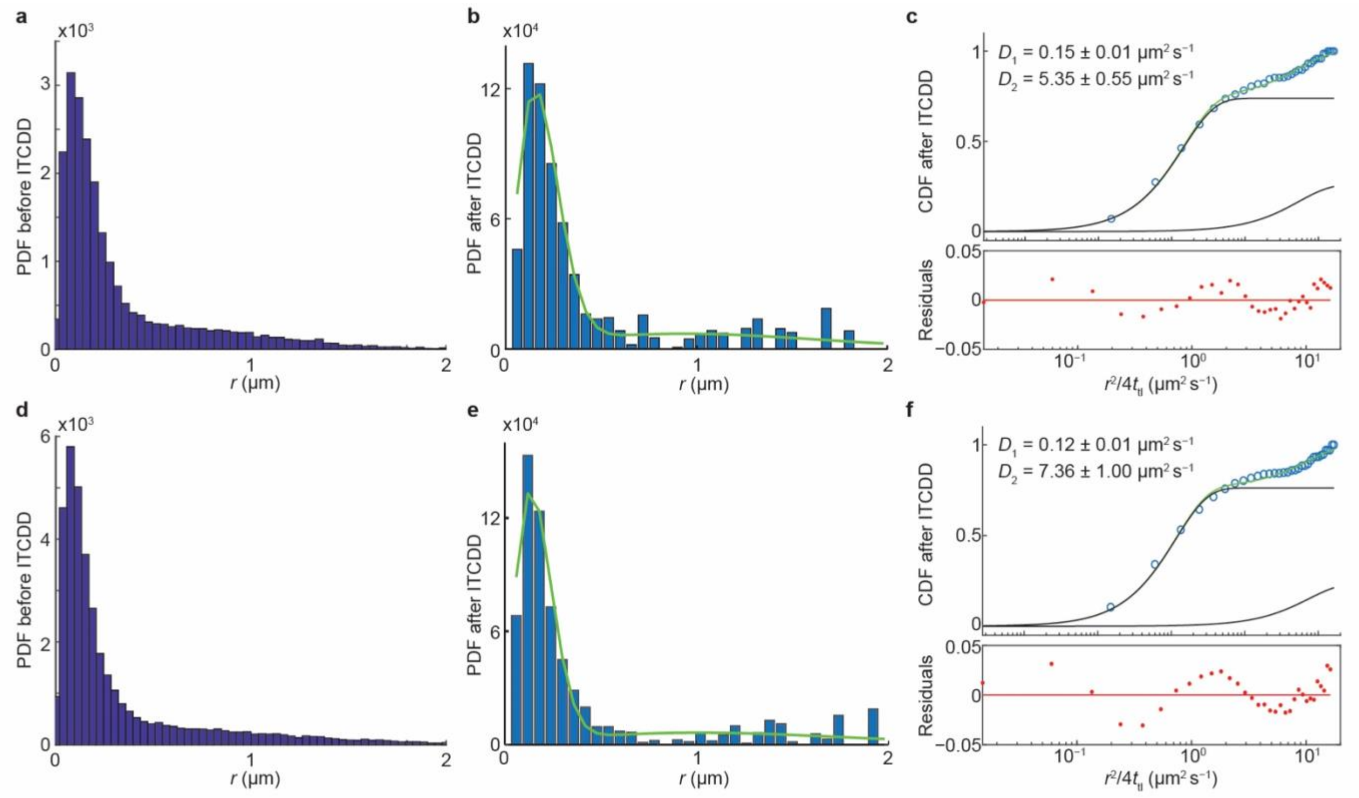

Supplementary Fig. 15 | Analysis of $CymA^{PmC}$ diffusion dynamics in *S. oneidensis*. a. The unnormalized PDF(r) before ITCDD correction for aerobically grown $CymA^{PmC}$. **b**, The unnormalized PDF(r) after ITCDD correction for aerobically grown $CymA^{PmC}$. Green curve: fitted results from the cumulative distribution function of displacement length, CDF(r) as in C. **c,** Fitting of CDF(r) after ITCDD resolves two diffusion states for aerobically grown CymA. Residual of fitting is shown in red dots; red line: zero line for eye guide. **d-f**, Same analysis for $CymA^{PmC}$ grown anaerobically with lactate and ferric citrate. Source data are provided as a Source Data file.

12.2 Determination of diffusion states of CymA in the cell from ITCDD-corrected CDF

To determine the minimal number of diffusion states of CymA, we further integrated the ITCDD-transformed PDF(r) into its corresponding cumulative distribution function (CDF) for analysis with multi-state Brown diffusion state models (Equation S3 for 2-state, where T_{tl} is the time lapse in our stroboscopic imaging protocol), as described previously[7]. Diffusion constants and fractional populations were extracted from the CDF fitting equation. Aerobically grown $CymA^{PmC}$ could be resolved into two distinct diffusion states (Supplementary Fig. 15c), one dominant mobile state with $D_1 = 0.15 \pm 0.01$ μm²/s, and another minor mobile state with $D_2 = 5.35 \pm 0.55$ μm²/s, with the corresponding fractional population $A_1 = 0.74 \pm 0.02$ and $A_2 = 0.26 \pm 0.02$, where $A_2 = 1 - A_1$. $CymA^{PmC}$ grown anaerobically with lactate and ferric citrate also could be resolved into two distinct diffusion states (Supplementary Fig. 15f), one dominant mobile state with $D_1 = 0.12 \pm 0.01$ μm²/s, and another minor mobile state with $D_2 = 7.36 \pm 0.55$ μm²/s, with the corresponding fractional population $A_1 = 0.76 \pm 0.02$ and $A_2 = 0.24 \pm 0.02$. All fitted results of CymA's diffusion constants, and its fractional populations grown either aerobic or aerobic conditions are summarized in Supplementary Table 4. The overall fitted results were also plotted on the ITCDD-corrected PDF(r) (Supplementary Fig. 15b and e, green line). Note that CymA puncta exhibited no discernible diffusion (with an apparent diffusion coefficient of 0.002 ± 0.001 μm²/s, i.e., effectively stationary) during the same period of CymA SMT acquisition (1.8 s for each molecule), thus the corrected single-molecule CymA diffusion coefficient remains effectively unchanged considering the error bars (Supplementary Fig. 16).

$$\mathrm{CDF}_{2\ \mathrm{states}} = A_1(1-\exp\left(-\frac{r^2}{4D_1T_{\mathrm{tl}}}\right)) + A_2(1-\exp\left(-\frac{r^2}{4D_2T_{\mathrm{tl}}}\right)) \quad \text{Equation S3}$$

Supplementary Table 4 | Diffusion constants and fractional populations of CymA grown under various growth conditions.

	Growth condition	D_1 (μm²/s)	A_1	D_2 (μm²/s)	A_2	copy number	[CymA] (μM)	No. of cells
**1**	AA/O_2	0.15 ± 0.01	0.74 ± 0.02	5.35 ± 0.55	0.26 ± 0.02	2952 ± 1002	4.8 ± 1.8	269
**2**	Lactate/Ferric citrate	0.12 ± 0.01	0.76 ± 0.02	7.36 ± 0.55	0.24 ± 0.02	3770 ± 1506	5.6 ± 2.6	481
**3**	Fe/Fumarate	0.10 ± 0.01	0.80 ± 0.02	5.37 ± 0.83	0.20 ± 0.02	2900 ± 1680	5.2 ± 2.8	272
**4**	Lactate/Ferric oxide	0.10 ± 0.01	0.82 ± 0.01	4.29 ± 0.59	0.18 ± 0.01	5512± 2360	6.6 ± 2.6	121
**5**	H_2/Fumarate	0.15 ± 0.01	0.78 ± 0.02	7.67 ± 1.16	0.22 ± 0.02	4802 ± 1970	4.6 ± 1.4	167

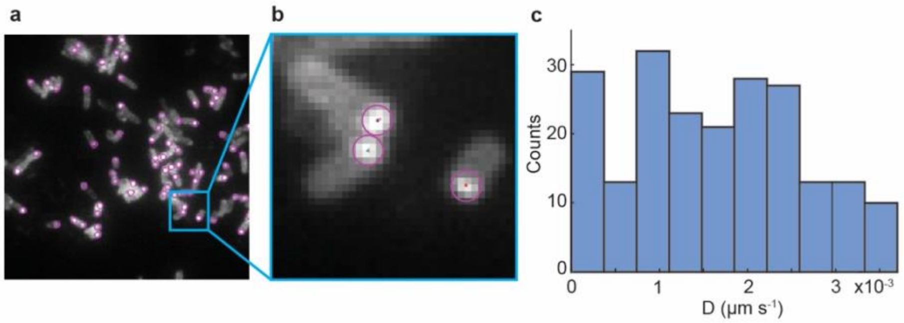

Supplementary Fig. 16 | CymA^{PmC} puncta are effectively stationary in *S. oneidensis*. a. Representative fluorescent image of CymA^{PmC} in *S. oneidensis* grown anaerobically with iron/fumarate, overlaid with tracked puncta (purple circle). b, Magnified view of selected cells with tracks in the puncta. **c,** Histogram of the apparent diffusion coefficient *D* of CymA^{PmC} puncta. A total of 209 puncta were tracked by open-source image J plugin, TrackMate[58] Mean ± s.d. = 0.002 ± 0.001 μm²/s. Source data are provided as a Source Data file.

13. Reconstruction of super-resolution images from single-molecule tracking trajectories

13.1 Reconstruction of single molecule tracking trajectories to super-resolution images sorted by displacement

Utilizing single-molecule tracking trajectories, we determined that CymA contains two diffusion state (Supplementary Notes 12.2, Supplementary Fig. 17a). To further investigate spatial localization of proteins within these two states, we classified approximately the tracking trajectories into two types of diffusions based on the magnitude of the first displacement: (1) classified as being part of the D_1 state, i.e., the dominant slow diffusion state CymA^{PmC}, if the first displacement length is smaller than a threshold value r_0; or (2) as being part of the D_2 state, i.e., the minor fast diffusion state CymA^{PmC}, if the first displacement length exceeds the threshold r_0. The value of r_0 is calculated as:

$$CDF(r,t) = 1 - exp\left(-\frac{{r_0}^2}{4Dt}\right) = 0.99 \quad \text{Equation S4}$$

Here, D is the diffusion constant of the D_1 in the original, distorted PDF, and t is the image time lapse (60 ms). This D is derived from a calibration curve of D values before and after ITCDD correction, which, as previously reported[19], was initially underestimated by a factor of 1.7 before ITCDD.

For CymA^{PmC} grown under aerobic conditions, the D_1 state after ITCDD is ~0.15 μm^2/s (Supplementary Table 4), corresponding to ~ 0.088 μm^2/s before ITCDD. This translates to r_0 = 311 nm according to Equation S4 (vertical yellow dashed line in Supplementary Fig. 17a), encompassing 99% of D_1 state displacement lengths. We examined the initial position of CymA^{PmC} associated with the D_1 slow diffusion state (Supplementary Fig. 17b) and D_2 fast diffusion state (Supplementary Fig. 17c), respectively. Both CymA^{PmC} were homogeneously located at the cell envelope, further supporting our assignments based on diffusion constants, where CymA molecules in the D_1 state represent inner membrane-anchored fraction and those in the D_2 state denote periplasmic fraction.

Similarly, we investigated spatial localization of CymA^{PmC} grown under anaerobic conditions with lactate/ferric oxide. D_1 state after ITCDD is ~0.10 μm^2/s (Supplementary Table 4), corresponding to ~ 0.059 μm^2/s before ITCDD. This yields r_0 = 255 nm according to Equation S4 (vertical yellow dashed line in Supplementary Fig. 17d), encompassing 99% of D_1 state displacement lengths. Analogously, we examined the initial position of CymA^{PmC} associated with the D_1 slow diffusion state (Supplementary Fig. 17e) and the D_2 fast diffusion state (Supplementary Fig. 17f), respectively. We observed that CymA^{PmC} in the D_1 state were primarily located at the punctum, while CymA^{PmC} in the D_2 state exhibited largely homogeneous distribution at the cell envelope. This suggests that CymA^{PmC} in the inner membrane are prone to punctum formation, thereby corroborating our hypothesis that raft-like lipid domains, formed with menaquinone and the membrane, are involved in the formation of CymA^{PmC} punctum. Additionally, it also further suggests against the possibility that self-interactions among CymA^{PmC} molecules are the drivers of their punctum formation, which, if were true, should lead to CymA^{PmC}'s periplasmic fraction to form puncta as well.

To quantify the topological distribution, we examined the distribution of pairwise distances between the first locations of each tracked CymA dominantly in D_1 state and D_2 state, respectively. For CymA^{PmC} grown under aerobic conditions, the mean values of pairwise distance distribution from locations dominantly in the D_1 state (inner-membrane-anchored fraction) and D_2 state (periplasmic fraction) were 400 and 470 nm, respectively (Supplementary Fig. 17d). On the other hand, for CymA^{PmC} grown under anaerobic conditions, the mean values of pairwise distance distribution of dominantly in the D_1 and D_2 states are 270 and 450 nm, respectively, showing a much larger difference (Supplementary Fig. 17h).

Therefore, the slower D_1 diffusion state (CymA in the membrane) changes their spatial distribution from aerobic growth to the anaerobic growth during EET, while the faster D_2 diffusion state (CymA in the periplasm) does not change their spatial distribution. These results indicate that CymA's spatial distribution changes are likely linked to membrane reorganization, which we proposed/hypothesized to be raft-like domain formation, due to menaquinone level increase in the membrane, which led to the formation of CymA^{PmC} puncta.

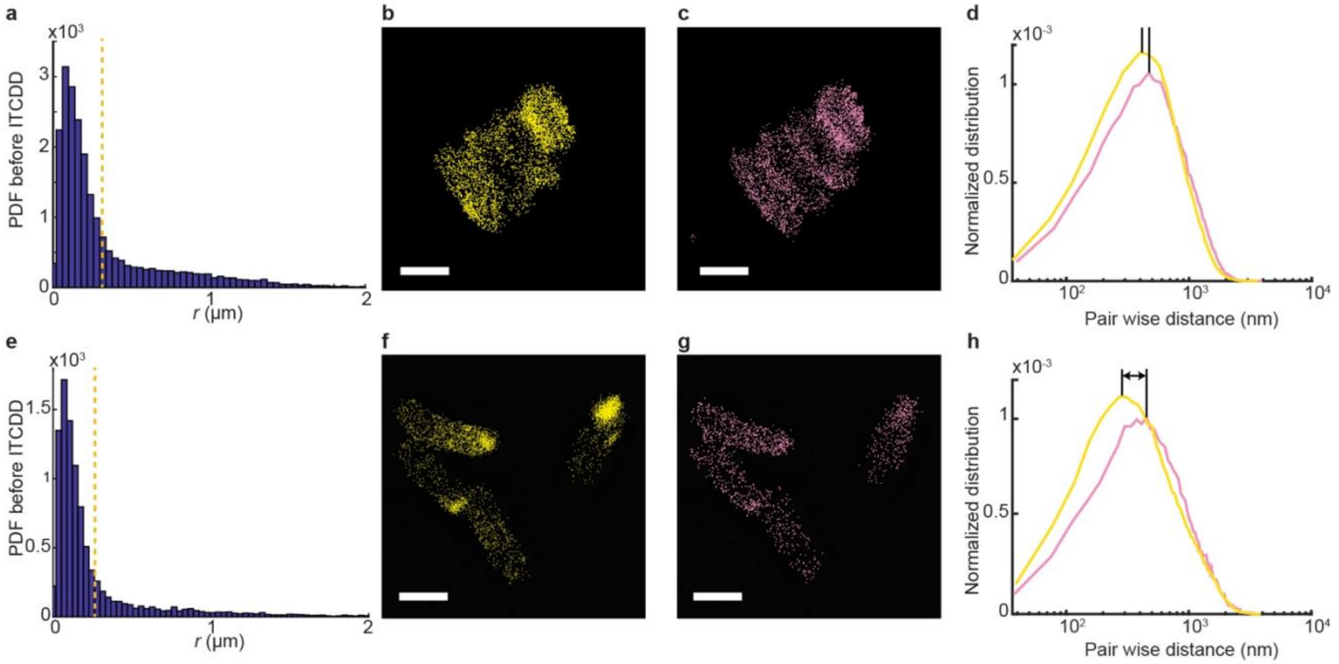

Supplementary Fig. 17 | Reconstructed super-resolution images of CymA in two diffusion states. a,e, PDF(r) of first displacement lengths from the CymA^{PmC} strain (a) grown under aerobic conditions, with a dashed yellow line marking the threshold (r_0) at 311 nm, and (e) grown under anaerobic conditions (Lactate/Ferric oxide), with the threshold at 255 nm. Displacement length r lower than the threshold encompass 99% of the dominant D_1 state (slow diffusion state) displacements in the original PDF(r). (a) and (e) are same figures as Supplementary Fig. 15a and d. **b, c, f, g,** Reconstructed super-resolution images of CymA^{PmC} molecules that are dominantly in the (b, f) D_1 diffusion state or dominanrly in the (c, g) D_2 diffusion state, corresponding to (b, c) aerobic and (f, g) anaerobic growth conditions. (f) is the same figure as Fig. 5d in the main text. Scale bars = 1 μm. **d,** Pairwise distance distribution of first locations of single CymA^{PmC} tracks shown in panel b (yellow line) and c (pink line). **h,** same as d but the tracked CymA^{PmC} molecules corresponding to panel f (yellow line) and g (pink line). Black vertical lines, mean value of the distribution. Source data are provided as a Source Data file.

13.2 CymA molecules either within or outside puncta have similar mobility.

We analyzed the diffusion trajectories of CymA^{PmC} molecules within and outside puncta separately. We collected the first displacement length r of CymA^{PmC} spatially located in the puncta, judged by the initial location of the displacement (Supplementary Fig. 19a), and those outside the puncta (Supplementary Fig. 19b). To have enough statistics, we combine all first displacements of CymA^{PmC} grown under anaerobic conditions (i.e., using lactate/ferric citrate, lactate/ferric oxide, metal iron/fumarate, hydrogen/fumarate, or cathode/fumarate as terminal electron donor/acceptor pairs).

Both displacement distributions comprise two distinct diffusion states as we observed, a dominant slow mobile state (D ~ 0.13 μm^2/s) and a minor fast mobile state (D ~ 4-7 μm^2/s), which are consistent across any other conditions (Supplementary Table 4). For CymA^{PmC} molecules located within the puncta are dominantly (fractional population ~84%) in slow state, with only a small fraction of fast mobile state (~16%). Similarly, CymA^{PmC} located outside the puncta displayed dominantly slow state (~73%), with a bit larger fractional populations of fast mobile state (~27%) compared with CymA^{PmC} located within the puncta. As we assigned in the main text, this minor fast-moving fraction represents periplasmic population of CymA^{PmC}, which is distributed across the cells and cannot be identified by merely their first locations being within or outside of the puncta due to the limitations of 2D projection imaging. The larger fractional population (~27% vs. ~16%) from the analysis of two displacement length distribution results from the fact that the area outside the puncta is generally larger than the area of puncta in the cell. The area ratio between puncta and the rest in cells is estimated as ~0.13 and it agrees with the ratio of CymA molecules of fast mobile state in puncta to outside puncta ~0.10 (a total of 20,273 CymA were quantified across 268 cells, with 2,918 localized within puncta and 17,355 outside puncta).

Therefore, CymA^{PmC} in the same dominant slow mobile state, assigned to inner-membrane protein, indeed exists in both regions (i.e., within or outside puncta), indicating that puncta/condensate formation does not slow down CymA diffusivity.

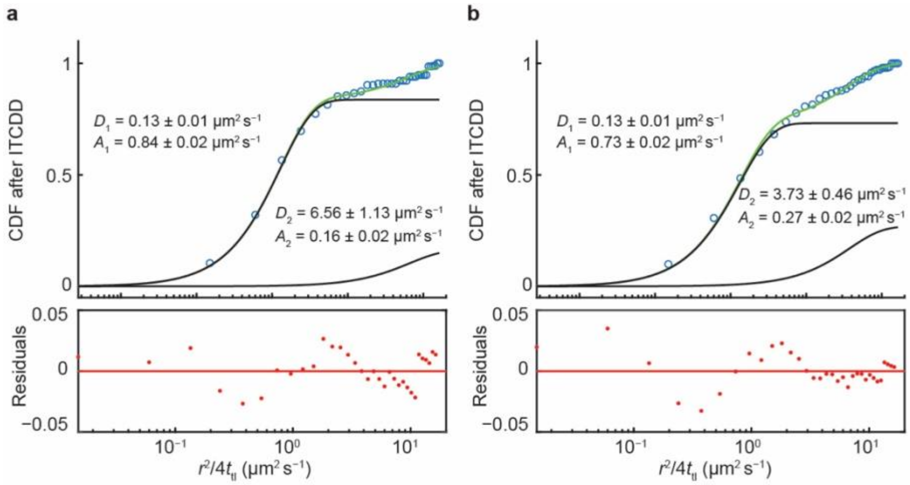

Supplementary Fig. 18 | CymA diffusivity does not change either within and outside puncta/condensate. a,b, Fitting of CDF(r) after ITCDD resolves two diffusion states for anaerobically grown CymA^{PmC} (Supplementary Notes 12.2). Displacement length r of CymA located (**a**) in condensate and those located (**b**) outside condensate were analyzed. Blue circles: data. Green curves: overall fitted results. Black lines: two resolved diffusion states. Residual of fitting is shown in red dots; red line: zero line for eye guide. Source data are provided as a Source Data file.

14. Kinetics of CymA protein moving out or into CymA puncta

14.1 Determination of escaping kinetics of CymA out of puncta

To determine whether and how fast CymA reversibly moving out of and into punctum, we examined the kinetics by approximating it as a simple two-state process: one state is CymA resides inside punctum and the other outside (i.e., free) (Equation S5), where k_e is the effective rate constant of CymA escaping the punctum, and k_c is the effective rate constant of forming punctum.

$$free \underset{k_e}{\overset{k_c}{\rightleftarrows}} condensate \qquad \text{Equation S5}$$

To determine k_e, first, we obtained the position and the radius of a punctum by fitting with a circle as described in Supplementary Notes 2 (Supplementary Fig. 19a-c). Then, we extracted the trajectories of a single CymA^{PmC} molecule's distance from the punctum center (x, y) using the single-molecule tracking results (Fig. 5g in the main text; Supplementary Fig. 19d). Thresholding these distance trajectories with the radii of the puncta (R) allowed for extracting individual CymA^{PmC}'s residence time, τ_{in}, in the punctum.

The distribution of τ_{in} was fitted with a single exponential function (Equation S6; Supplementary Fig. 19e; N is a scaling parameter) with k_{eff} being the exponential decay rate constant, which contains k_{bl}, the photobleaching/blinking rate constant of PmC, and k_e, the effective escaping rate constant of CymA out of the punctum (Equation S7). k_{bl} was independently determined from analyzing the distribution of the fluorescence on-times in the tracking trajectories (Supplementary Fig. 19f), as described in our previous work[17]. As our imaging uses a stroboscopic illumination scheme with time lapse in between images, the laser pulse duration (t_{int}) 4 ms and time lag (t_{tl}) 60 ms are include in affecting k_{bl} as in Equation S7. The kinetic values are summarized in Supplementary Table 6.

$$y = Nexp(-k_{eff}\tau) \qquad \text{Equation S6}$$

$$k_{eff} = k_{bl}\frac{t_{int}}{t_{tl}} + k_e \quad \text{Equation S7}$$

To probe how the radii threshold R may affect the extracted escaping kinetics, we fitted a punctum, as an alternative, with an elliptical instead of a circle to have an upper (RU) and lower bound (RL) of radius (Supplementary Fig. 19c). With different thresholds (i.e., RU and RL), the corresponding escaping rate constants k_e were calculated, resulting in $k_{e,ub}$ or $k_{e,lb}$ being 5.2 s^{-1} or 4.0 s^{-1}, respectively; the numbers didn't change much, with k_e being approximately 3-5 s^{-1}.

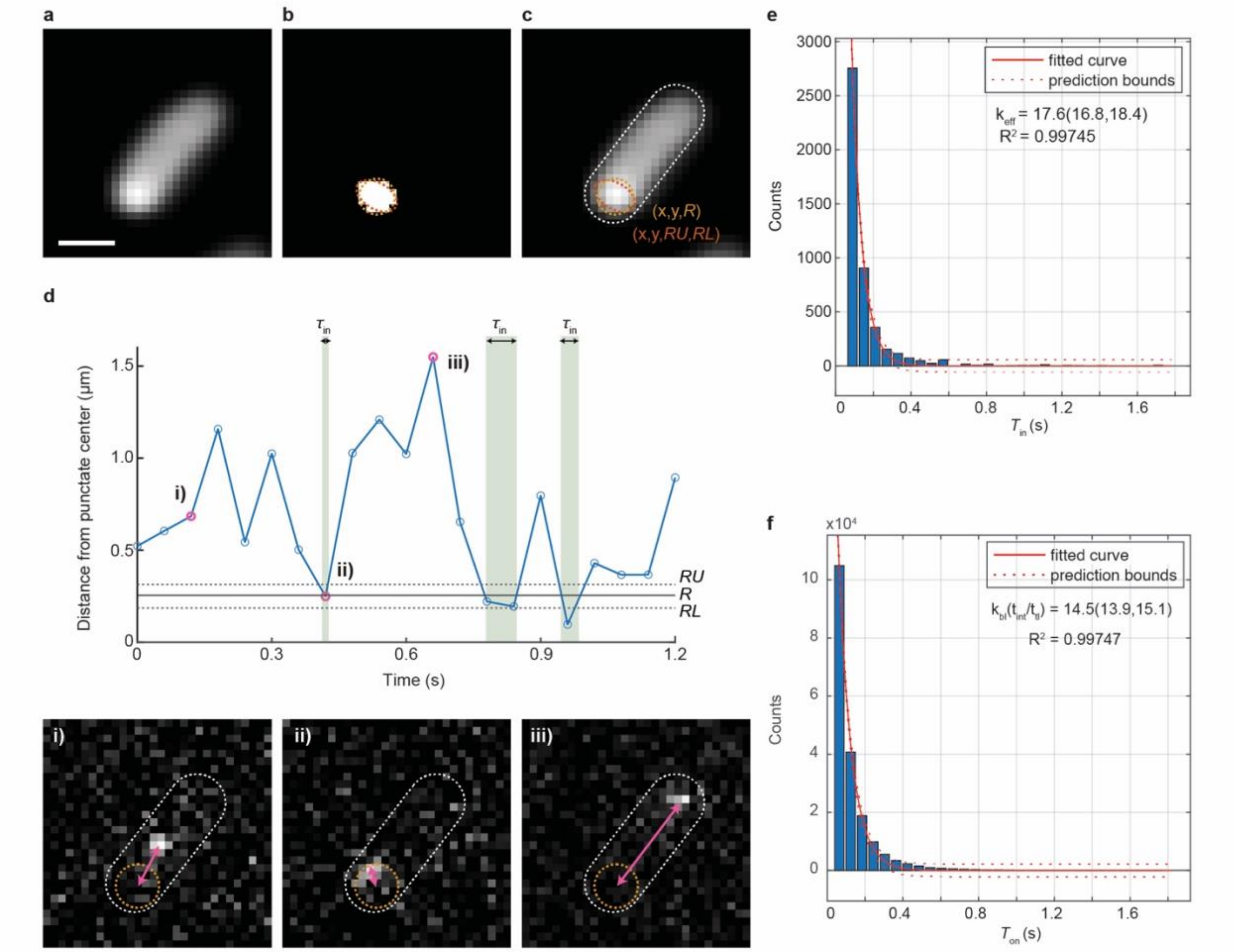

Supplementary Fig. 19 | Determination of escaping kinetics of CymA from punctum. a, Ensemble-activated fluorescence image of $CymA^{PmC}$ cells grown anaerobically with lactate and ferric citrate. Scale bar = 1 μm. **b,** Binary image from (**a**) illustrating puncta classified by the machine learning algorithm, with the punctum fitted with either a circle (yellow dashed line) or an ellipse (red dashed line). **c,** Same image as (**a**), with cell contour using a fitted hemisphere-capped-rod model (white dashed line) and punctum contour using a fitted circle or ellipse. By fitting a circle, the center position of a punctum (x, y) and radius (R) were obtained, and by fitting an ellipse, the center position of a punctum (x, y), major axis radius (RU), and minor axis radius (RL) were obtained. **d,** Upper: Trajectory of the distance between a single $CymA^{PmC}$ molecule and a punctum center. The residence time (τ_{in}) of the molecule inside the punctum is thresholded by R (dashed line). Lower: Three exemplary images showing the distance (pink arrow) between a single CymA molecule and the punctum center, denoted in the trajectory plot (i-iii), were present. **e,** Distribution of $CymA^{PmC}$'s residence time τ_{in} in puncta, where the decay rate constant is k_{eff}. 4563 residence times (τ_{in}) were collected from 346 cells. **f,** Distribution of the fluorescence on-times (τ_{on}) of $CymA^{PmC}$ in the tracking trajectories, where the decay rate constant gives the photobleaching/blinking rate constant k_{bl} of PmC (t_{int} = 0.04, t_{tl} =

0.60). 551024 fluorescence on-times (τ_{on}) were collected from 346 cells were collected from Solid lines in **e-f**: single exponential fit with 95% confidence bounds (dotted lines). Source data are provided as a Source Data file.

14.2 Determination of effective rate constant of forming puncta via equilibrium approximation

Once the effective escaping rate constant, k_e was determined, we further estimated the effective condensation rate constant k_c of CymA, using the approximate 2-state model (Equation S5) and the CymA populations located within a condensate (A_c) and outside of a condensate (A_f) (Equation S8):

$$k_c = k_e \times \frac{A_c}{A_f} \qquad \text{Equation S8}$$

To obtain the ratio A_c/A_f of CymA between two distinguished locations, we employed two distinct methods: (1) comparing the integrated fluorescence intensity within and outside of condensate in the cell, utilizing fluorescence images where all PmC-tagged CymA molecules were excited (SCQPC in Supplementary Methods 1.5); (2) Counting the number of individual CymA molecules imaged during single-molecule tracking, where the initial positions of CymA were used for location determination.

In both methods in determining the values of A_c and A_f,, we calculated both the mean and weighted mean among individual cells (based on the fluorescence intensity or the number of CymA) to avoid biases stemming from cells with exceptionally high or low copy numbers. During the calculation, outlier beyond the 99th percentile were excluded. The resulting A_c/A_f ratios are summarized in Supplementary Table 5. With the obtained ratios, the effective condensation rate constant k_c of CymA is estimated to be ~0.6 s^{-1}. All kinetic values are summarized in Supplementary Table 6.

Supplementary Table 5 | Ratio of CymA populations located within puncta (A_c) and outside of puncta (A_f) in individual cells. CymA^{PmC} grown under anaerobic conditions. All anaerobic condition results were combined to derive the parameters.

A_c/A_f	Mean ± s.d.	Weighted mean ± s.d.
**Method (1)**	0.18 ± 0.30	0.20 ± 0.33
**Method (2)**	0.19 ± 0.13	0.17 ± 0.13

Supplementary Table 6 | Kinetic parameters of CymA grown under anaerobic conditions. All anaerobic condition results were combined to derive the parameters.

k_{bl} (s^{-1})	k_{eff} (s^{-1})	k_e (s^{-1})	$k_{e,lb}$ (s^{-1})	$k_{e,ub}$ (s^{-1})	k_c (s^{-1})
217.7 ± 9.0[a]	17.6 ± 0.8[a]	3.1 ± 1.4[b]	4.0 ± 1.4[b]	5.2 ± 1.5[b]	0.6 ± 1.3[c]

[a] Errors are 95% confidence intervals.
[b] Errors are determined with error propagation from k_{bl} and k_{eff} based on equation S7.
[c] Error are determined with error propagation from k_e and A_c/A_f based on equation S8. With all A_c/A_f in Supplementary Table 5, errors are 0.65-1.3.

15. Additional supplementary figures

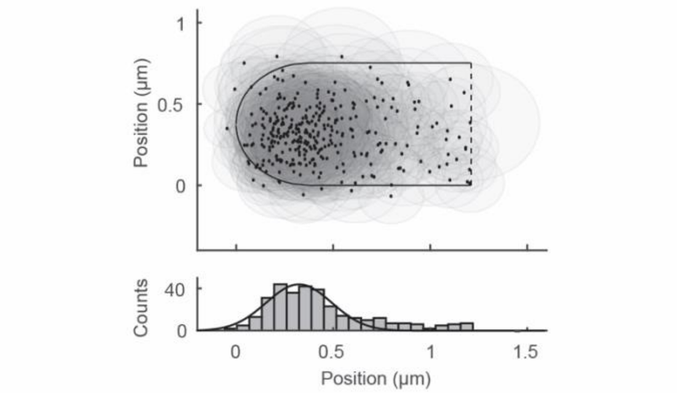

Supplementary Fig. 20 | Locations of CymA^{PmC} puncta in the cell. Top: Locations of CymA^{PmC} punctum centers (black dots) and sizes (approximated as grey circles; Supplementary Notes 2) (sample size $n = 346$) overlaid on half of the average cell geometry (black lines). Some black dots appear outside the average geometry because of some larger cells. Bottom: Projected histogram of punctum center positions along the cell length, with a Gaussian fit centered at 0.33 μm (fwhm = 0.40 μm). Source data are provided as a Source Data file.

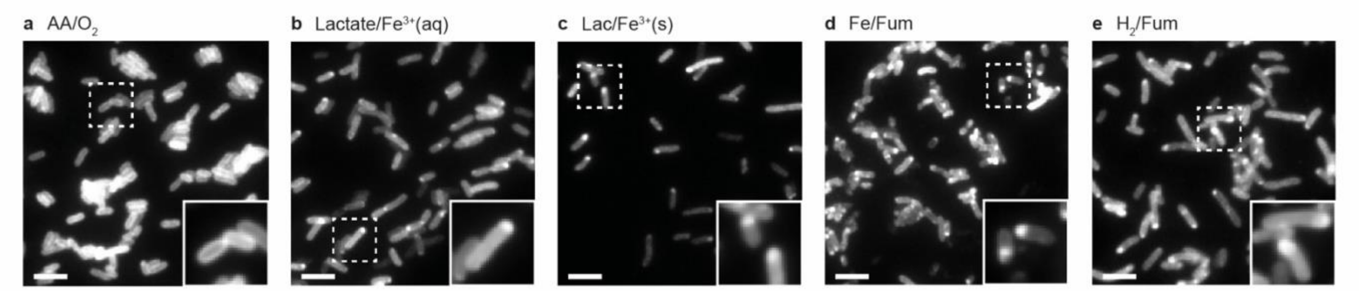

Supplementary Fig. 21 | Additional wide-field fluorescence images of ensemble-photoactivated CymA^{PmC} in *S. oneidensis* grown under aerobic and anaerobic conditions. a-e, Cells were grown with different electron donor/terminal electron acceptor pairs as labelled above the images; these conditions are mentioned in Fig. 1f in the main text. Cells showed punctate pattern when engaged in EET under anaerobic growth. Insets: zoom-in of dotted boxes. Scale bars: 5 μm.

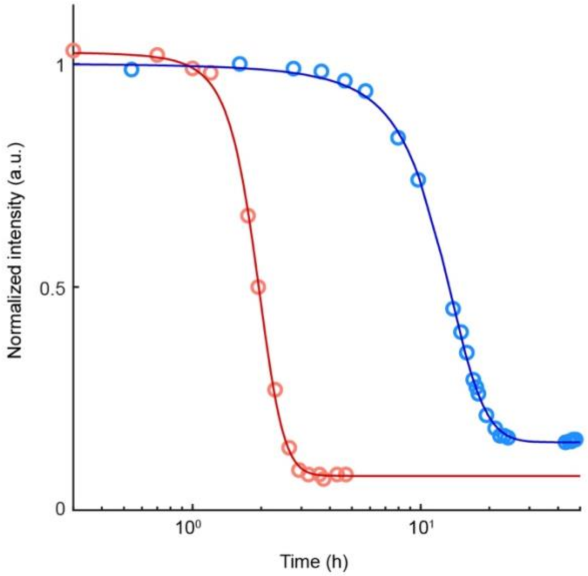

Supplementary Fig. 22 | Time course of redox activity by quasi-wild-type *S. oneidensis*, from analyzing data extracted from literature[4,59]. Quasi-wild-type *S. oneidensis* oxidizes 5 mM AHDS with 5 mM fumarate terminal electron acceptors (red circle). Quasi-wild-type mutants contain a transposon insertion but have no effect on $AHDS_{red}$ oxidation. As $AHDS_{red}$ is oxidized to

AQDS$_{ox}$ by cells, the medium color changes from yellow-orange to clear. Yellow intensity was calculated and normalized. We fitted the data to a sigmoidal function to extract the lag time and transition time, which are 1.3 ± 0.1 h and 1.1 ± 0.1 h, respectively (red line, errors: s.e.). The original data and detailed method are described in the previous paper[4]. Quasi-wild-type *S. oneidensis* reducing 5 mM AQDS with 5 mM lactate electron donor (blue circle). As the AQDS dye is reduced and becomes orange, the intensity of the blue channel dropped. Blue channel intensity was calculated and normalized. A lower blue intensity indicates greater reduction of the dye. We fitted the data points to sigmoidal function to extract the lag time and transition time, which are 6 ± 0.1 h and 12 ± 0.3 h, respectively (blue line, errors: s.e.). The original data and detailed method are described in the previous paper[59]. Source data are provided as a Source Data file.

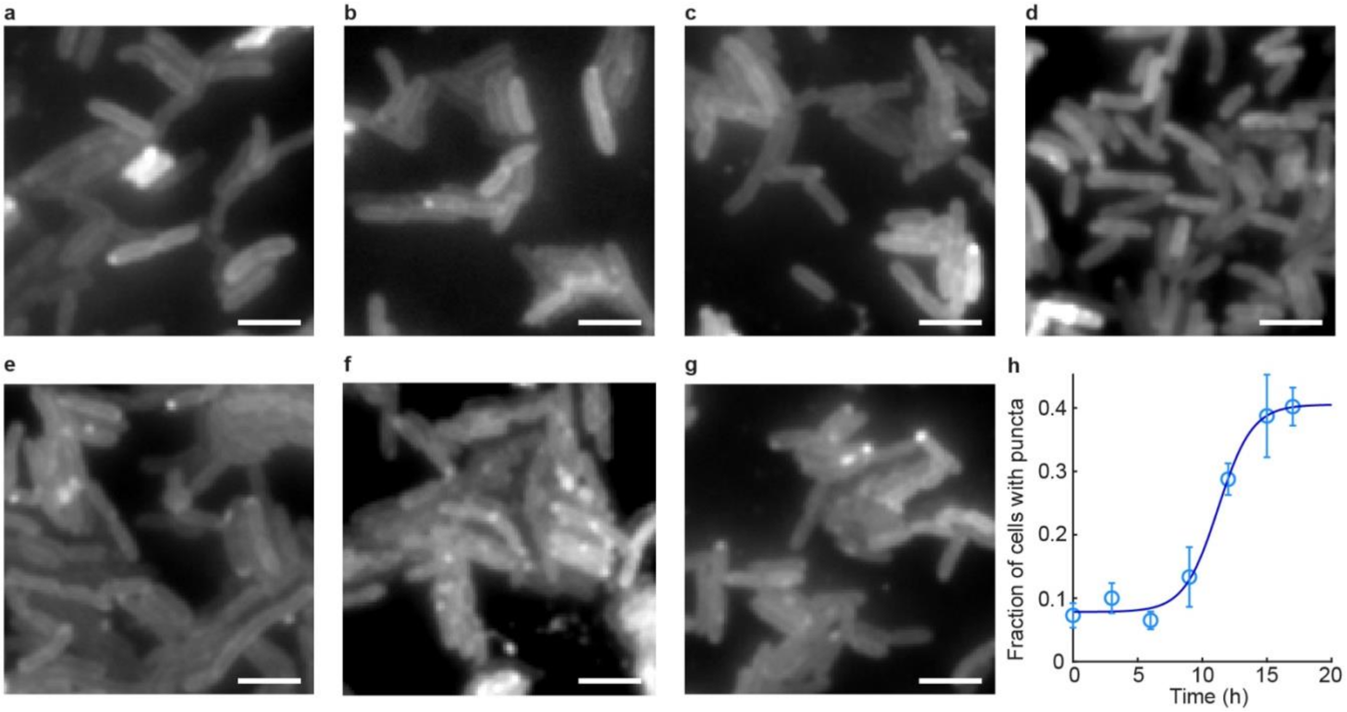

Supplementary Fig. 23 | CymA punctum formation was observed much slower on an electrode with applied anodic potential for electron outflow. a-g, Ensemble fluorescence images of CymA^{PmC} in *S. oneidensis* while applying potentials (+0.60 V vs. Ag/AgCl). Images were captured (**a**) 0 h, (**b**) 3 h, (**c**) 6 h, (**d**) 9 h, (**e**) 12 h, (**f**) 15 h, (**g**) 17 h after potential application. **h,** Fraction of cells exhibiting CymA^{PmC} puncta vs. time. Means and error bars (s.d.) are from 2-3 biological replicates with the calculations weighed by the cell counts. Lines: sigmoidal fits. Extracted lag time is 9 ± 1 h and transition time is 5 ± 1 h (errors: s.e.). Different time points are from different sets of cells (number of images: 4,3,3,3,2,3,2; total number of cells: 423, 280, 377, 466, 76, 266, 75 for a total of 7 time points in increasing order). Lines: fits with a sigmoidal function (Supplementary Notes 4). Scale bars: 3 μm. Source data are provided as a Source Data file.

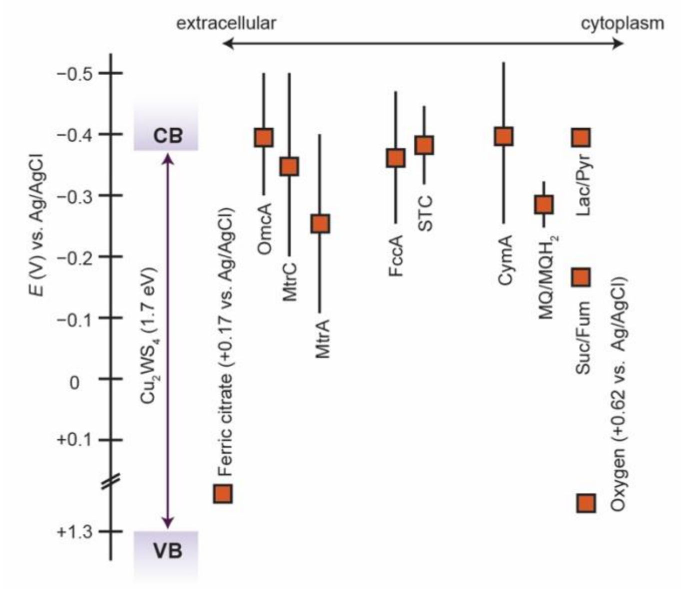

Supplementary Fig. 24 | Reduction Potentials of the selected EET proteins in *S. oneidensis* and the energies of conduction and valence band edges of Cu_2WS_4. Cu_2WS_4, excitable by 405 nm laser, has a conduction band edge sufficiently high to reduce proton in water (−0.2 V vs. Ag/AgCl) and OM electron-transfer proteins (−0.2 to −0.4 V vs. Ag/AgCl). The reduction potentials of proteins are from the previous reports[60–66]. MQ/MQH_2: menaquinone/menaquinol, Lac: lactate, Pyr: pyruvate, Suc: succinate, Fum: fumarate.

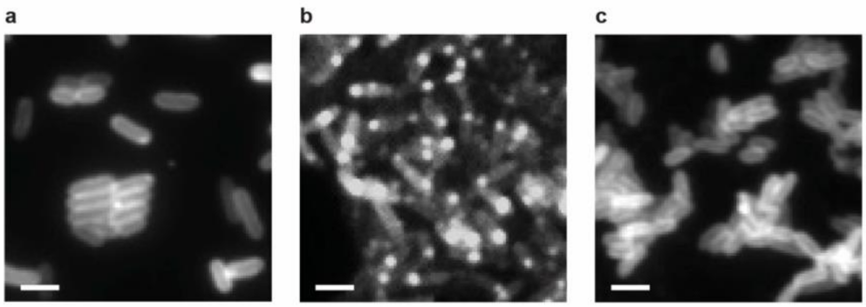

Supplementary Fig. 25 | CymA punctum formation is driven by EET on an electrode. a-c, Ensemble fluorescence images of CymA^{PmC} *S. oneidensis* cells grown aerobically and then placed on ITO-coated glass slide. Images were taken (**a**) before and (**b**) 5 h after applying potentials (–0.48 V vs. Ag/AgCl), and cells (**c**) 5 h after without applying potentials as a control. *S. oneidensis* did not form CymA puncta without applying potential, indicating that punctum formation is indeed driven by EET, not by contact with electrode or limited nutrient. Scale bars = 2 μm.

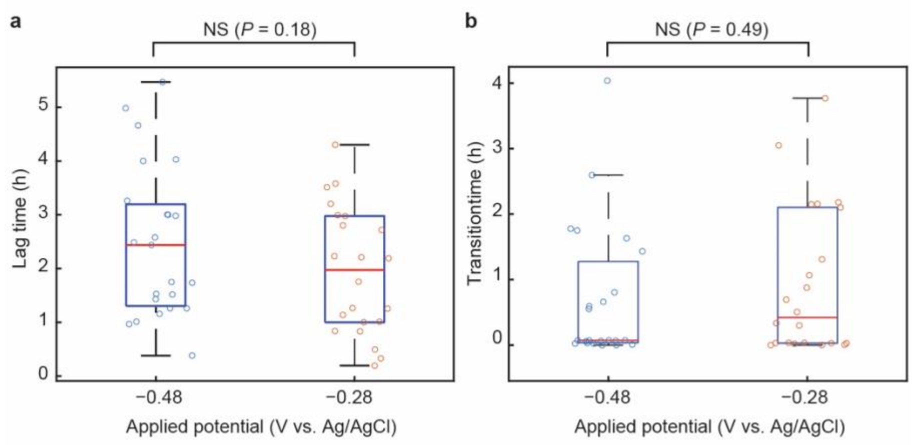

Supplementary Fig. 26 | Changes in electron-transfer driving force using different electrochemical potentials does not cause discernible difference in single-cell CymA punctum formation dynamics. CymA^{PmC} *S. oneidensis* cells were grown aerobically and placed on ITO-coated glass slide. Then potential (–0.28 V vs. Ag/AgCl) was applied. We observed similar CymA punctum formation as when more negative potential (–0.48 V vs. Ag/AgCl) was applied as presented Fig. 2 in the main text. **a,b,** With the same analysis (Supplementary Notes 5), there was no significant differences in (**a**) lag time and (**b**) transition time. n = 29, 29. Statistical significance was determined using Welch's two-sample t-test (NS, not significant; $P < 0.05$, significant). Box plots were shown with median and 1.5 IQR whiskers. Source data are provided as a Source Data file.

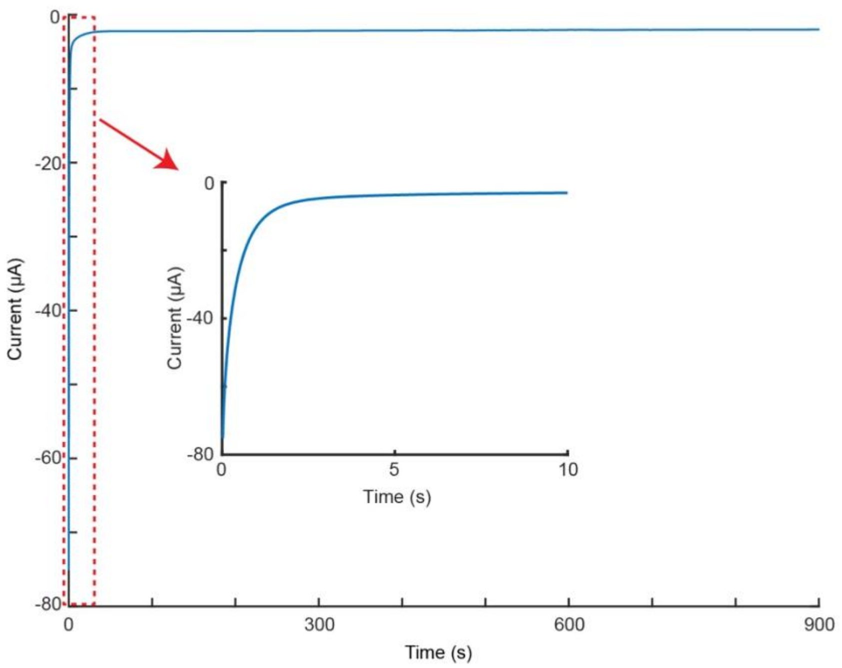

Supplementary Fig. 27 | Chronoamperometric data at a constant poised cathodic potential. Current vs. time at a constant –0.48 V vs. Ag/AgCl corresponding to Fig. 2. Due to the potentiostat's hardware/software limitations, current was measured for 900 s per run, and the poising potential was reapplied repeatedly for a total duration of 6 h. Inset: a magnified view of the first 10 s, highlighting the initial current drift and subsequent stabilization. Source data are provided as a Source Data file.

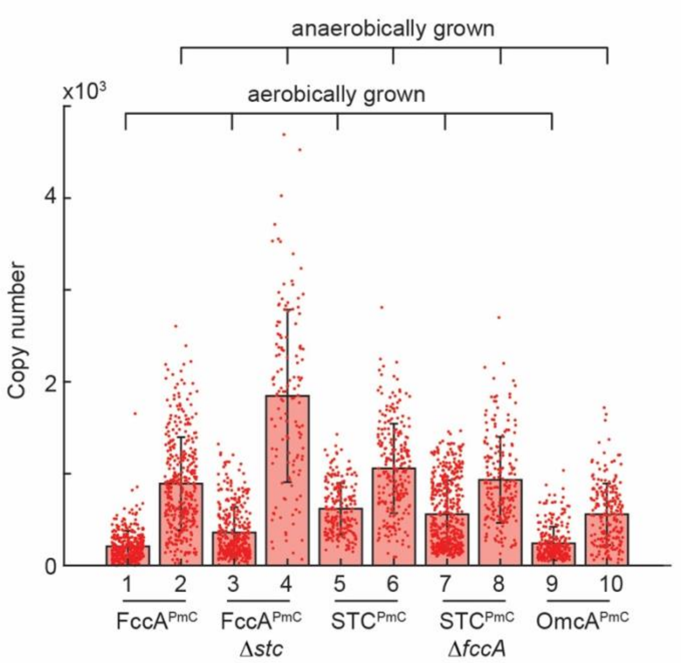

Supplementary Fig. 28 | Periplasmic and outer membrane EET proteins in *S. oneidensis* were up-regulated under anaerobic growth (lactate/ferric citrate). a-c, Copy numbers of periplasmic proteins (FccA and STC) and outer membrane protein (OmcA) significantly increased. All five strains were significantly upregulated (****; $P < 0.0001$) after anaerobically growth (1, 3, 5, 7, 9 vs. 2, 4, 6, 8, 10). Statistical significance was determined using Welch's two-sample *t*-test (NS, not significant; $P < 0.05$, significant). Mean (bar) and s.d. (error bars). (sample size n = 338, 357, 353, 133, 203, 248, 458, 200, 256, 207). Source data are provided as a Source Data file.

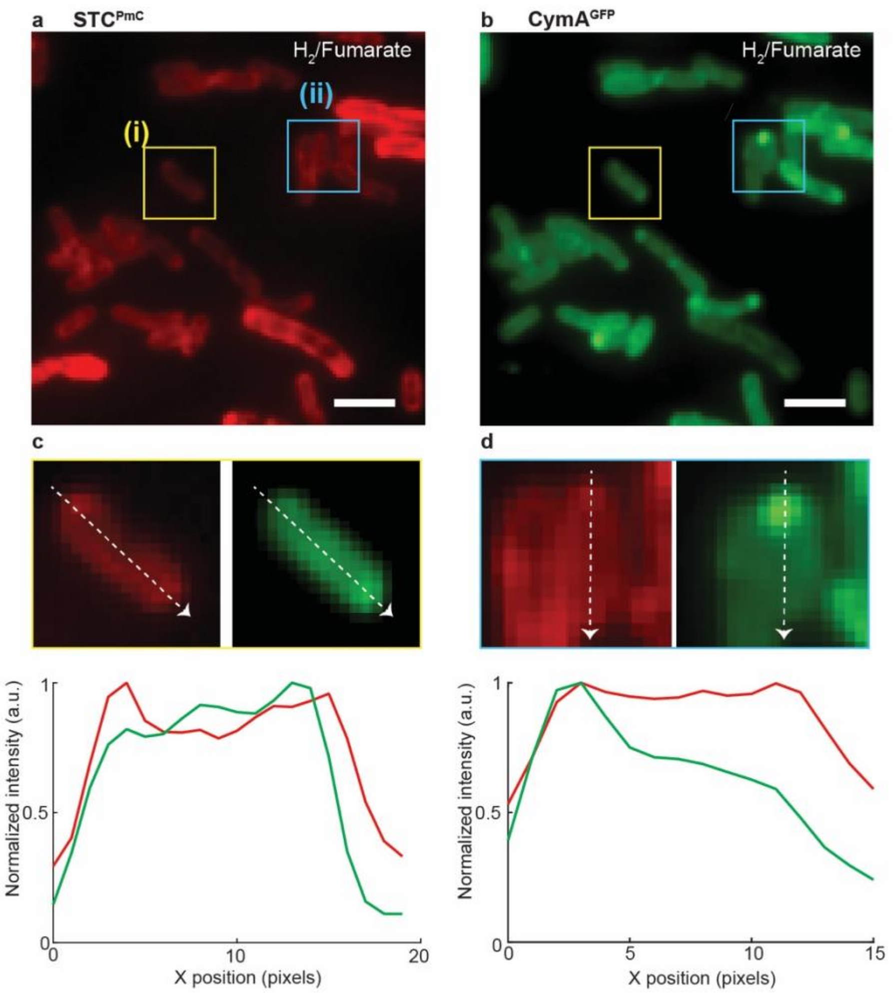

Supplementary Fig. 29 | Intensity line profile analysis shows clearly that STC is homogeneously distributed when not involved in EET, while CymA forms puncta. a,b, Fluorescence images of ensemble-photoactivated STC^{PmC} (a) or $CymA^{GFP}$ (b) in cells grown anaerobically with hydrogen/fumarate as the electron donor/acceptor pair where EET does not involve STC. These figures are replicate of Fig. 3 c-d in the manuscript. Scale bar = 5 µm. **c, d,** Zoom-in image of the yellow box (i) and blue box (ii) in a-b. White arrows denote the direction of line drawing. Normalized intensity line profile of STC^{PmC} (red line) showed apparent bipolar accumulation due to homogeneous membrane localization, while that of $CymA^{GFP}$ (green line) showed much higher intensity at one end due to puncta formation. These data clearly support that CymA punctum formation can occur independently from STC, indicating that STC is not the driver of CymA punctum formation. Source data are provided as a Source Data file.

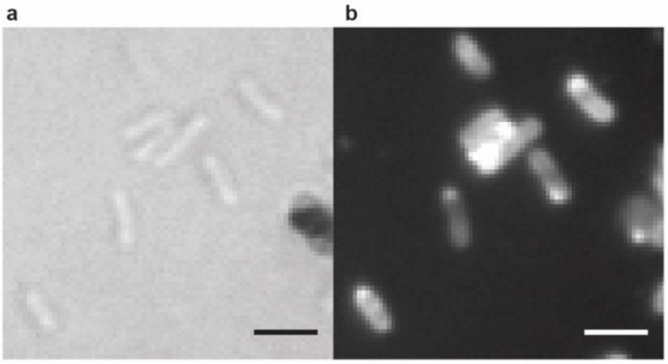

Supplementary Fig. 30 | $CymA^{GFP}$ is imageable by fluorescence atop Cu_2WS_4 film and formed puncta in cells under anaerobic growth as well as $CymA^{PmC}$. a, Transmission and **b,** fluorescence images of $CymA^{GFP}$ grown anaerobically (lactate/ferric citrate) for 2 days placed on Cu_2WS_4 film on ITO-coated glass slide. $CymA^{GFP}$ puncta are clearly visible in many cells on top of a semiconductor film, enabling us to differentiate cells that had or had not formed CymA puncta. Additionally, this

result showed that CymA puncta formation is not caused by the specific PmC tag, as CymA here is tagged with sfGFP. Scale bars = 2 μm.

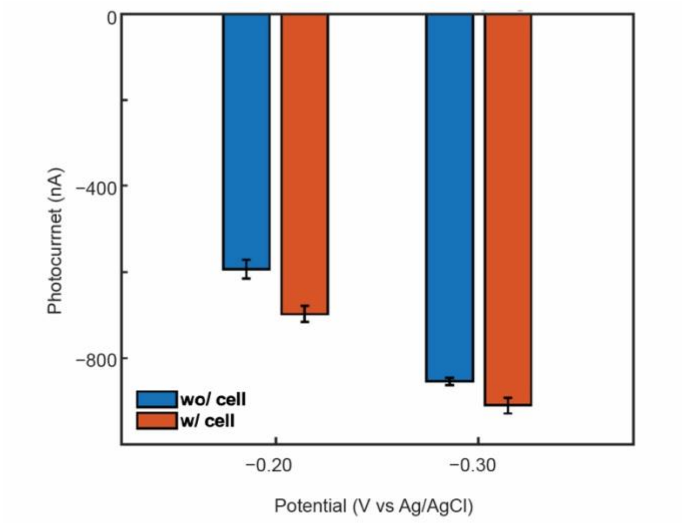

Supplementary Fig. 31 | Bulk photoelectrochemical measurements show cell-enhanced photo-cathodic current. *S. oneidensis* WT cells were grown anaerobically (lactate/ferric citrate) for 2 days and placed on Cu_2WS_4 thin-film on ITO-coated glass slide. Detailed measurement procedure is in Supplementary Methods 1.8. We observed cell-enhanced photo-cathodic current, indicating that cells can uptake electrons from semiconductor *directly*. Source data are provided as a Source Data file.

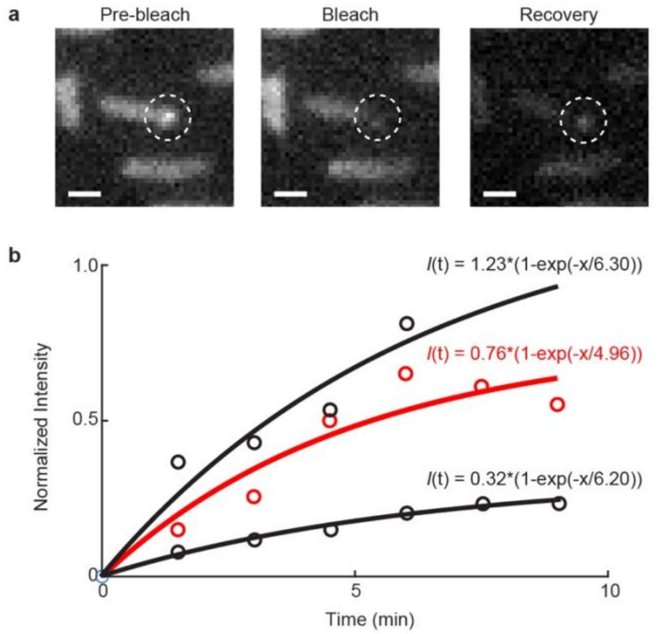

Supplementary Fig. 32 | Fluorescence recovery after photobleaching (FRAP) measurement. a, Representative epifluorescence *S. oneidensis* cells pre-grown anaerobically with lactate/ferric citrate. Using a laser beam of ~1 um in lateral size (dotted line), CymA puncta was photobleached for 10 s. Then, the fluorescence images were recorded at 90 s intervals for up to 9 min afterward. Scale bars: 1 μm. **b**, Three normalized fluorescence intensity trajectories, fitted with exponential function. The data in red line is the data shown in (a). The fluorescence in the bleached region recovered with a half time of 3.4 ± 2.2 min to approximately 76 ± 52% of the initial intensity at steady state, indicating that CymA molecules in puncta are mobile. The other two bleached regions recovered with half-times of 4.4 ± 4.2 min (upper black line) and 4.3 ± 2.4 min (lower black line), reaching ~123 ± 73% and ~32 ± 11% of their initial intensities, respectively. All errors are 95% confidence bounds from fits. Compared to single molecule tracking data, diffusion coefficient is much slower, but it might be due to the limited diffusion from the one side, taking longer time to recover, consistent with the previous study[67]. Source data are provided as a Source Data file.

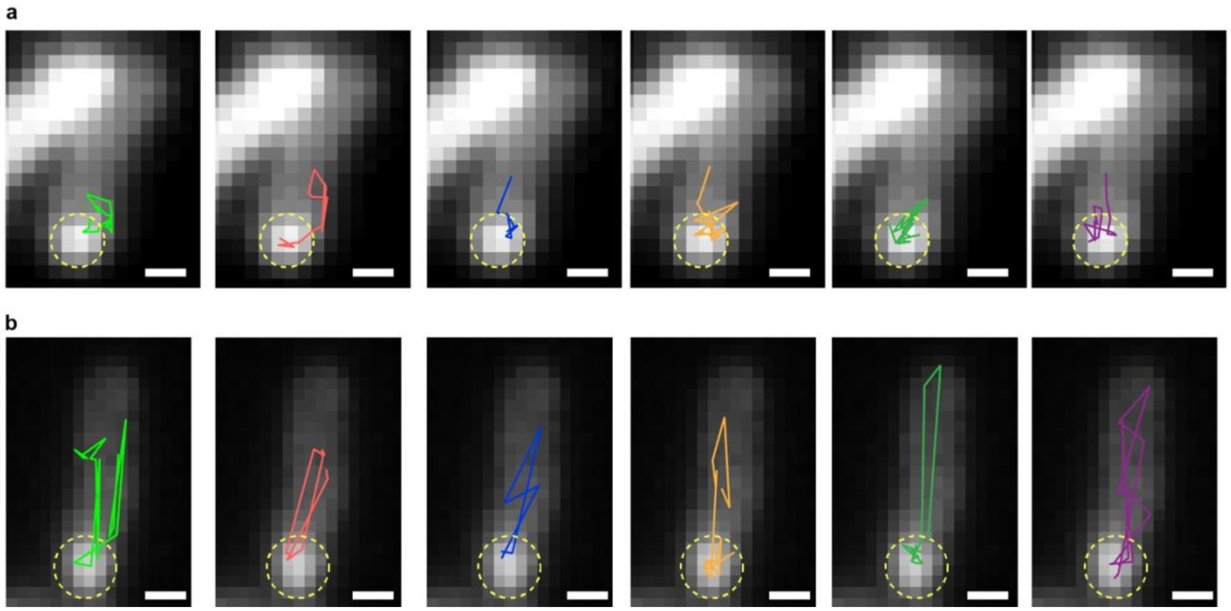

Supplementary Fig. 33 | Additional exemplary single-molecule tracking trajectories overlaid on wide-field fluorescence image of ensemble-photoactivated CymA^{PmC}. a,b, multiple single-molecule CymA^{PmC} tracks in two different cells move in/out of puncta indicating these puncta are membrane-less domains and exhibit dynamic exchange with the surrounding. Dashed yellow circle: puncta outline. Scale bars: 0.5 μm.

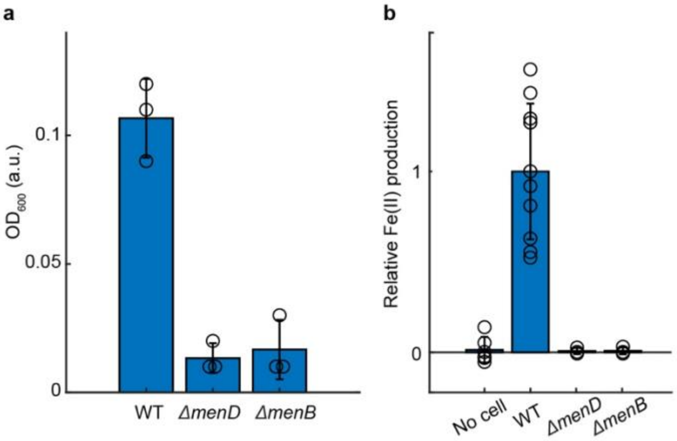

Supplementary Fig. 34 | Deletion mutation of menaquinone synthase is fetal to cell. a, OD_{600} of the growth culture after 2 days anaerobic growth in the medium supplemented with lactate and ferric citrate. Menaquinone synthase deletion mutants (Δ*menD* and Δ*menB*) showed growth disruption. **b,** Cells grown with the same condition as (a), Menaquinone synthase deletion mutants (Δ*menD* and Δ*menB*) could not reduce ferric citrate. Data were collected from multiple repeats. Mean ± error bars (s.d.). Source data are provided as a Source Data file.

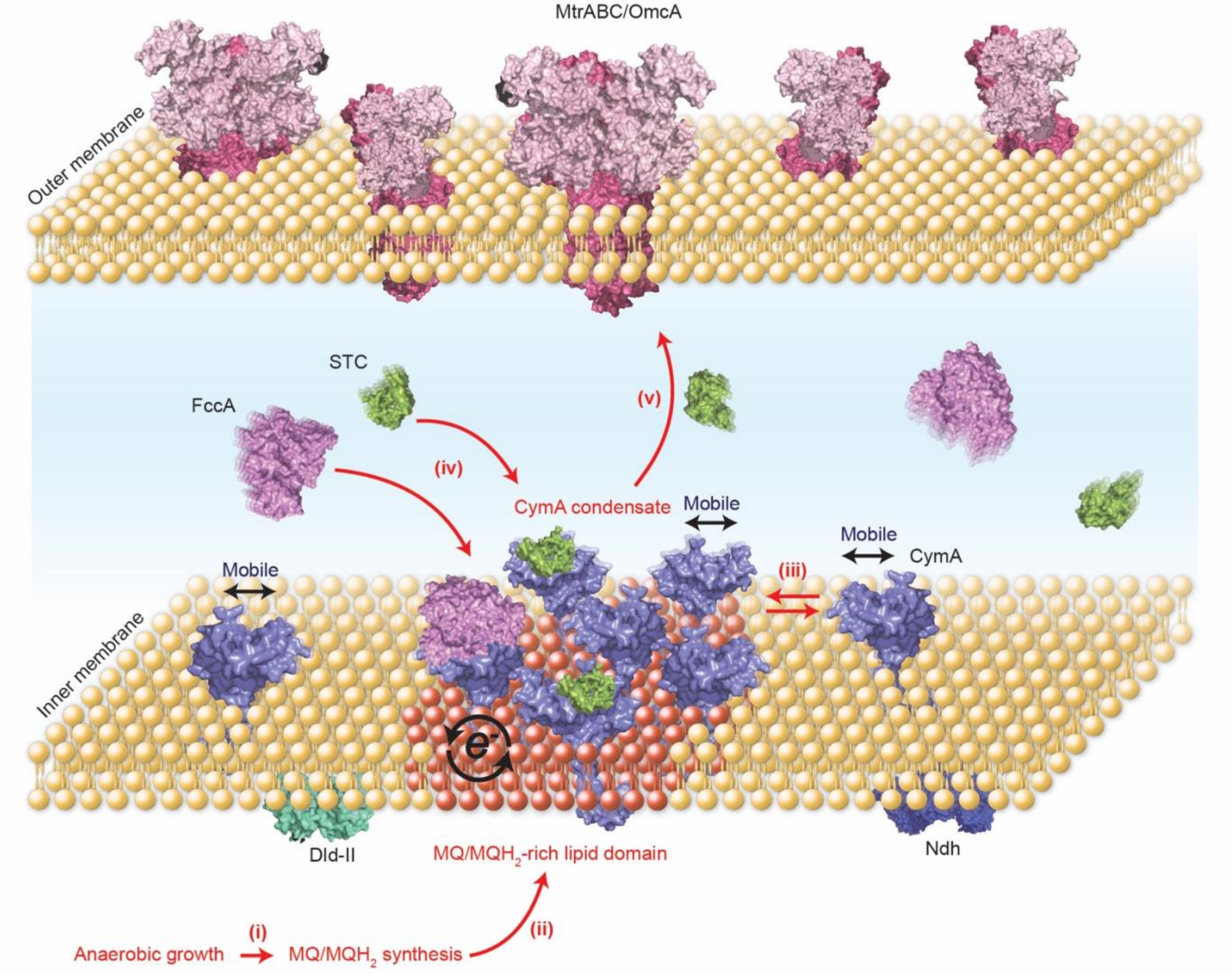

Supplementary Fig. 35 | Schematic of CymA punctum/condensate formation associated with inner-membrane domains enriched with menaquinone (MQ or MQH_2) and subsequent colocalization of periplasmic electron-transfer partners for EET. (i) During anaerobic growth, *S. oneidensis* upregulates the anaerobic electron carrier menaquinone. (ii) Then, menaquinones and polar lipids form raft-like microdomains in the inner membrane. (iii) CymA dynamically forms biomolecular condensate associating with lipid domains enriched in menaquinones, in which CymA maintains its mobility. This facilitates the electron exchange between CymA and menaquinone; the latter can exchange electrons with inner-membrane proteins like Dld-II and Ndh. (iv) CymA's direct electron-transfer partners, STC and FccA, in the periplasm approach CymA in the condensate and interact dynamically with CymA through direct protein-protein interaction[27,68] to exchange electrons as part of EET pathways, showing colocalization. (v) STC and FccA can subsequently dissociate from the condensate (Supplementary Fig. 37) and diffuse toward the outer-membrane to exchange electrons with MtrABC/OmcA complex[27]. Structures shown here from either crystal structure or homology modeling: FccA (PDB: 1QJD[9]); STC (PDB: 6EE7[10]); MtrABC (PDB: 6R2Q[8]); OmcA (PDB: 4LMH[11]); Structures of CymA, Dld-II and Ndh are predicted by AlphaFold[13,14].

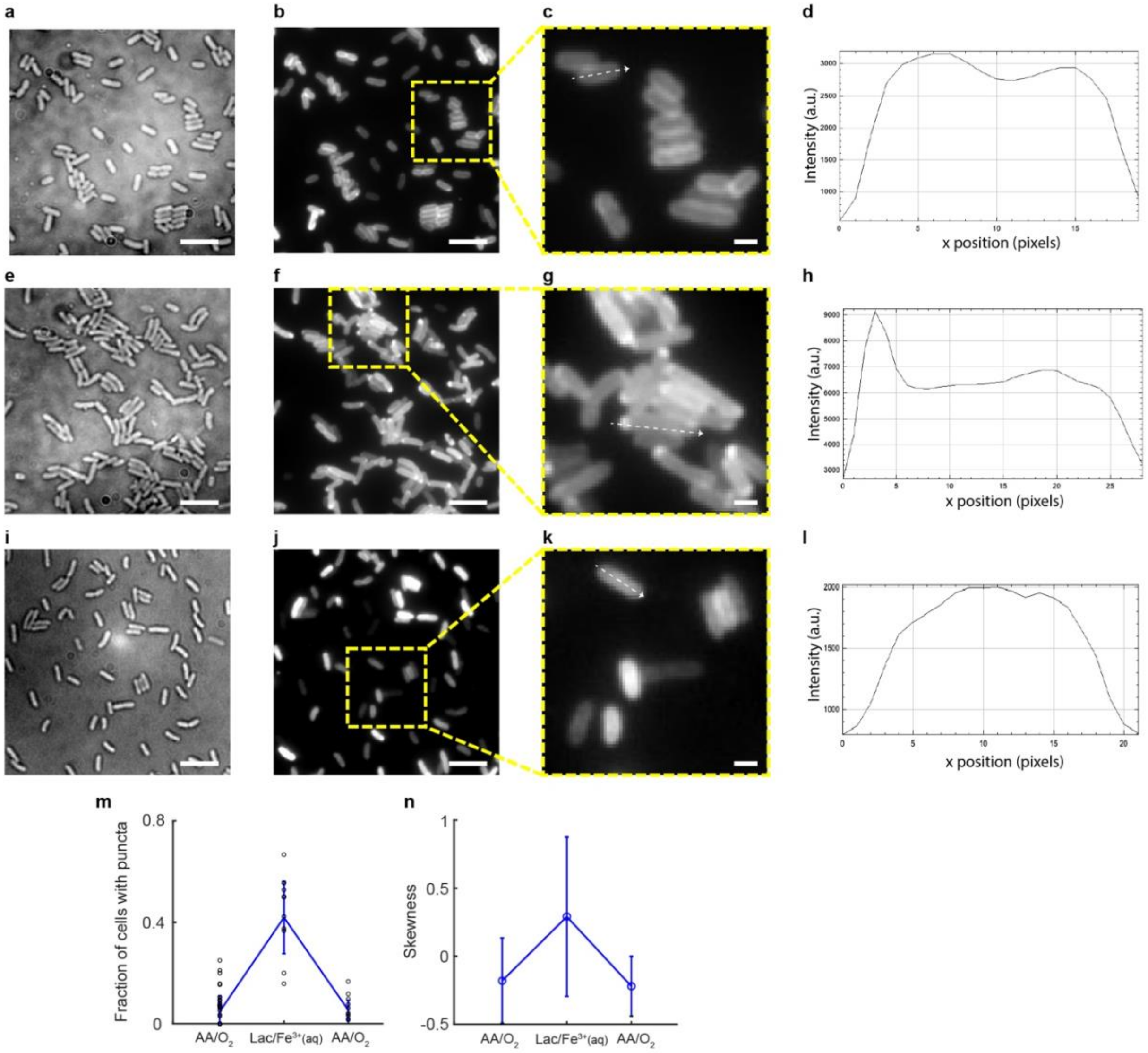

Supplementary Fig. 36 | When anaerobically grown cells with CymA^{PmC} puncta were re-introduced to aerobic conditions, CymA became uniformly distributed across the cell envelope. a-d, (a) Transmission image and (b) wild-field fluorescence image of CymA^{PmC} cells grown aerobically with amino acids and oxygen. (c) Zoom-in of dotted boxes in (b). (d) Fluorescence intensity profiles along the line marked by a white dashed arrow in (c). **e-h,** (e) transmission image and (f) wild-field fluorescence image of CymA^{PmC} cells grown anaerobically with lactate and ferric citrate, exhibiting CymA puncta. (g) Zoom-in of dotted boxes of (e). (h) Fluorescence intensity profiles along the line marked by a white dashed arrow in (g). **i-l,** (i) Transmission image and (j) wild-field fluorescence image of anaerobically grown CymA^{PmC} cells re-introduced to aerobic condition and grow for 8 h. CymA puncta disappeared and CymA became uniformly distributed across the cell envelope. (k) Zoom-in of dotted boxes of (j). (l) Fluorescence intensity profiles along the line marked by a white dashed arrow in (k). Note that we observed that membrane localization of CymA somewhat decreased after returning to aerobic condition. Scale bars: 3 μm. **m,** Fraction of cells showing CymA^{PmC} puncta grown condition corresponding to (a-d), (e-h), and (i-l) for each column. 1st and 2nd column data are same as Fig. 1f. For 3rd column, total 462 cells in different 10 images were counted. Open circles: fractions of cells with puncta from individual images. Means and error bars (s.d.) are from multiple images from 2-3 biological replicates, with the calculations weighed by the cell counts. **m,** Skewness corresponding to three conditions in (n). Means (open circle) and error bars (s.d.). Source data are provided as a Source Data file.

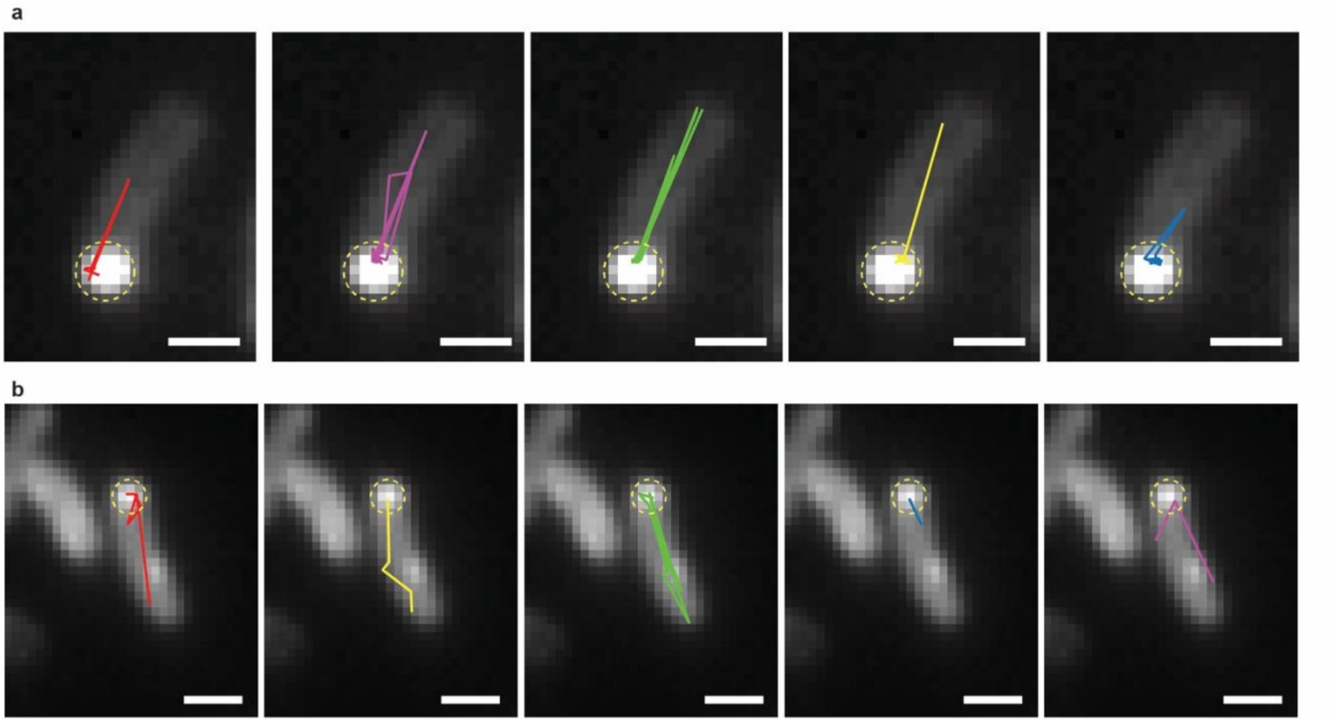

Supplementary Fig. 37 | Dynamic interactions of STC/FccA with CymA condensates. a,b, Representative single-molecule tracking trajectories (colored lines) overlaid on wide-field fluorescence images of ensemble-photoactivated (a) STC^{PmC} and (b) $FccA^{PmC}$. These single-molecule trajectories show periplasmic proteins moving into and out of condensates, supporting dynamic association and dissociation of STC/FccA with CymA condensate. Dashed yellow circle: condensate outline. Scale bars: 1 μm.

Supplementary references

1. Tang, Y. J., Meadows, A. L., Kirby, J. & Keasling, J. D. Anaerobic central metabolic pathways in *Shewanella oneidensis* MR-1 reinterpreted in the light of isotopic metabolite labeling. *J. Bacteriol.* **189**, 894–901 (2007).
2. Ringø, E., Stenberg, E. & Strøm, A. R. Amino acid and lactate catabolism in trimethylamine oxide respiration of *Alteromonas putrefaciens* NCMB 1735. *Appl. Environ. Microbiol.* **47**, 1084–1089 (1984).
3. Serres, M. H. & Riley, M. Genomic analysis of carbon source metabolism of *Shewanella oneidensis* MR-1: predictions versus experiments. *J. Bacteriol.* **188**, 4601–4609 (2006).
4. Rowe, A. R. *et al.* Identification of a pathway for electron uptake in *Shewanella oneidensis*. *Commun. Biol.* **4**, 957 (2021).
5. Yong, Y., Yu, Y., Zhang, X. & Song, H. Highly active bidirectional electron transfer by a self-assembled electroactive reduced-graphene-oxide-hybridized biofilm. *Angew. Chem. Int. Ed.* **53**, 4480–4483 (2014).
6. Saltikov, C. W. & Newman, D. K. Genetic identification of a respiratory arsenate reductase. *Proc. Natl. Acad. Sci. USA* **100**, 10983–10988 (2003).
7. Fu, B. *et al.* Single-cell multimodal imaging uncovers energy conversion pathways in biohybrids. *Nat. Chem.* **15**, 1400–1407 (2023).
8. Edwards, M. J., White, G. F., Butt, J. N., Richardson, D. J. & Clarke, T. A. The crystal structure of a biological insulated transmembrane molecular wire. *Cell* **181**, 665-673.e10 (2020).
9. Taylor, P., Pealing, S. L., Reid, G. A., Chapman, S. K. & Walkinshaw, M. D. Structural and mechanistic mapping of a unique fumarate reductase. *Nat. Struct. Biol.* **6**, (1999).
10. Huang, J. *et al.* Mesoscopic to macroscopic electron transfer by hopping in a crystal network of cytochromes. *J. Am. Chem. Soc.* **142**, 10459–10467 (2020).

11. Edwards, M. J. *et al.* The X-ray crystal structure of *Shewanella oneidensis* OmcA reveals new insight at the microbe–mineral interface. *FEBS Lett.* **588**, 1886–1890 (2014).
12. Rodrigues, M. L., Oliveira, T. F., Pereira, I. A. C. & Archer, M. X-ray structure of the membrane-bound cytochrome *c* quinol dehydrogenase NrfH reveals novel haem coordination. *EMBO J.* **25**, 5951–5960 (2006).
13. Jumper, J. *et al.* Highly accurate protein structure prediction with AlphaFold. *Nature* **596**, 583–589 (2021).
14. Varadi, M. *et al.* AlphaFold Protein Structure Database in 2024: providing structure coverage for over 214 million protein sequences. *Nucleic Acids Res.* **52**, D368–D375 (2024).
15. Edwards, R. A., Keller, L. H. & Schifferli, D. M. Improved allelic exchange vectors and their use to analyze 987P fimbria gene expression. *Gene* **207**, 149–157 (1998).
16. Stookey, L. L. Ferrozine-a new spectrophotometric reagent for iron. *Anal. Chem.* **42**, 779–781 (1970).
17. Chen, T.-Y. *et al.* Concentration- and chromosome-organization-dependent regulator unbinding from DNA for transcription regulation in living cells. *Nat. Commun.* **6**, 7445 (2015).
18. Jung, W., Sengupta, K., Wendel, B. M., Helmann, J. D. & Chen, P. Biphasic unbinding of a metalloregulator from DNA for transcription (de)repression in live bacteria. *Nucleic Acids Res.* **48**, 2199–2208 (2020).
19. Fu, B. *et al.* Metal-induced sensor mobilization turns on affinity to activate regulator for metal detoxification in live bacteria. *Proc. Natl. Acad. Sci. USA* **117**, 13248–13255 (2020).
20. Heckmeier, P. J. & Langosch, D. Site-specific fragmentation of green fluorescent protein induced by blue light. *Biochemistry* **60**, 2457–2462 (2021).
21. Durisic, N., Laparra-Cuervo, L., Sandoval-Álvarez, Á., Borbely, J. S. & Lakadamyali, M. Single-molecule evaluation of fluorescent protein photoactivation efficiency using an in vivo nanotemplate. *Nat. Methods* **11**, 156–162 (2014).
22. Chen, P. & Chen, T.-Y. MATLAB code package: iQPALM (image-based quantitative photo-activated localization microscopy). https://doi.org/10.6084/m9.figshare.12642617.v1. (2020).
23. Xie, F., Pei, S., Lin, X., Tian, Y. & Zhang, G. A rapid and efficient method for the extraction and identification of menaquinones from *Actinomycetes* in wet biomass. *BMC Microbiol.* **21**, 175 (2021).
24. Myers, J. M. & Myers, C. R. Role of the tetraheme cytochrome cyma in anaerobic electron transport in cells of *Shewanella putrefaciens* MR-1 with normal levels of menaquinone. *J. Bacteriol.* **182**, 67–75 (2000).
25. Li, N. *et al.* Charge separation in facet-engineered chalcogenide photocatalyst: a selective photocorrosion approach. *Nanoscale* **6**, 9695–9702 (2014).
26. Coursolle, D. & Gralnick, J. A. Modularity of the Mtr respiratory pathway of *Shewanella oneidensis* strain MR-1. *Mol. Microbiol.* **77**, 995–1008 (2010).
27. Fonseca, B. M. *et al.* Mind the gap: cytochrome interactions reveal electron pathways across the periplasm of *Shewanella oneidensis* MR-1. *Biochem. J.* **449**, 101–108 (2013).
28. Berg, S. *et al.* ilastik: interactive machine learning for (bio)image analysis. *Nat. Methods* **16**, 1226–1232 (2019).
29. Reid, B. G. & Flynn, G. C. Chromophore formation in green fluorescent protein. *Biochemistry* **36**, 6786–6791 (1997).
30. Zhang, C., Xing, X.-H. & Lou, K. Rapid detection of a gfp-marked *Enterobacter aerogenes* under anaerobic conditions by aerobic fluorescence recovery. *FEMS Microbiol. Lett.* **249**, 211–218 (2005).
31. Shi, L. *et al.* Isolation of a high-affinity functional protein complex between omca and mtrc: two outer membrane decaheme *c* -type cytochromes of *Shewanella oneidensis* MR-1. *J. Bacteriol.* **188**, 4705–4714 (2006).
32. Chong, G. W. *et al.* Single molecule tracking of bacterial cell surface cytochromes reveals dynamics that impact long-distance electron transport. *Proc. Natl. Acad. Sci. USA* **119**, e2119964119 (2022).

33. Subramanian, P., Pirbadian, S., El-Naggar, M. Y. & Jensen, G. J. Ultrastructure of *Shewanella oneidensis* MR-1 nanowires revealed by electron cryotomography. *Proc. Natl. Acad. Sci. USA* **115**, (2018).
34. Pirbadian, S. *et al. Shewanella oneidensis* MR-1 nanowires are outer membrane and periplasmic extensions of the extracellular electron transport components. *Proc. Natl. Acad. Sci. USA* **111**, 12883–12888 (2014).
35. Chong, G. W., Pirbadian, S. & El-Naggar, M. Y. Surface-induced formation and redox-dependent staining of outer membrane extensions in *shewanella oneidensis* mr-1. *Front. Energy Res.* **7**, 87 (2019).
36. Sivula, K. & Van De Krol, R. Semiconducting materials for photoelectrochemical energy conversion. *Nat. Rev. Mater.* **1**, 15010 (2016).
37. Nasir, J. A. *et al.* Recent developments and perspectives in CdS-based photocatalysts for water splitting. *J. Mater. Chem. A* **8**, 20752–20780 (2020).
38. Sambur, J. B. *et al.* Sub-particle reaction and photocurrent mapping to optimize catalyst-modified photoanodes. *Nature* **530**, 77–80 (2016).
39. Mao, X. & Chen, P. Inter-facet junction effects on particulate photoelectrodes. *Nat. Mater.* **21**, 331–337 (2022).
40. Nathan, C. & Cunningham-Bussel, A. Beyond oxidative stress: an immunologist's guide to reactive oxygen species. *Nat. Rev. Immunol.* **13**, 349–361 (2013).
41. Unden, G. & Bongaerts, J. Alternative respiratory pathways of Escherichia coli: energetics and transcriptional regulation in response to electron acceptors. *Biochim. Biophys. Acta* **1320**, 217–234 (1997).
42. Ksenzhek, O. S. & Petrova, S. A. Electrochemical properties of flavins in aqueous solutions. *J. Electroanal. Chem. Interf. Electrochem.* **156**, 105–127 (1983).
43. Dong, H. *et al.* Photocatalytic Cu_2WS_4 Nanocrystals for Efficient Bacterial Killing and Biofilm Disruption. *Int. J. Nanomedicine* **17**, 2735–2750 (2022).
44. Dai, Y. *et al.* Interface of biomolecular condensates modulates redox reactions. *Chem* **9**, 1594–1609 (2023).
45. Hu, K., Relton, E., Locker, N., Phan, N. T. N. & Ewing, A. G. Electrochemical Measurements Reveal Reactive Oxygen Species in Stress Granules**. *Angew. Chem. Int. Ed.* **60**, 15302–15306 (2021).
46. Dohnalkova, A. C. *et al.* Imaging Hydrated Microbial Extracellular Polymers: Comparative Analysis by Electron Microscopy. *Appl. Environ. Microbiol.* **77**, 1254–1262 (2011).
47. Liu, H., Newton, G. J., Nakamura, R., Hashimoto, K. & Nakanishi, S. Electrochemical characterization of a single electricity-producing bacterial cell of *Shewanella* by using optical tweezers. *Angew. Chem. Int. Ed.* **49**, 6596–6599 (2010).
48. McLean, J. S. *et al.* Quantification of electron transfer rates to a solid phase electron acceptor through the stages of biofilm formation from single cells to multicellular communities. *Environ. Sci. Technol.* **44**, 2721–2727 (2010).
49. Gross, B. J. & El-Naggar, M. Y. A combined electrochemical and optical trapping platform for measuring single cell respiration rates at electrode interfaces. *Rev. Sci. Instrum.* **86**, 064301 (2015).
50. Tang, Z. *et al.* Plasmonic probing single-cell bio-current waves with a shrinking magnetite nanoprobe. *ACS Nano* **16**, 20842–20850 (2022).
51. Jiang, X. *et al.* Probing single- to multi-cell level charge transport in *Geobacter sulfurreducens* DL-1. *Nat. Commun.* **4**, 2751 (2013).
52. Vellingiri, A. *et al.* Overexpression of c-type cytochrome, CymA in *Shewanella oneidensis* MR-1 for enhanced bioelectricity generation and cell growth in a microbial fuel cell. *J. Chem. Technol. Biotechnol.* **94**, 2115–2122 (2019).
53. Rosenbaum, M. A. *et al.* Transcriptional analysis of *Shewanella oneidensis* MR-1 with an electrode compared to Fe(III)citrate or oxygen as terminal electron acceptor. *PLoS ONE* **7**, e30827 (2012).
54. Charania, M. A. *et al.* Involvement of a membrane-bound class III adenylate cyclase in regulation of anaerobic respiration in *Shewanella oneidensis* MR-1. *J. Bacteriol.* **191**, 4298–4306 (2009).

55. Gao, H. *et al.* Physiological roles of ArcA, Crp, and EtrA and their interactive control on aerobic and anaerobic respiration in *Shewanella oneidensis*. *PLoS ONE* **5**, e15295 (2010).
56. Chen, T.-Y. *et al.* Quantifying multistate cytoplasmic molecular diffusion in bacterial cells via inverse transform of confined displacement distribution. *J. Phys. Chem. B* **119**, 14451–14459 (2015).
57. Oswald, F., L. M. Bank, E., Bollen, Y. J. M. & Peterman, E. J. G. Imaging and quantification of trans-membrane protein diffusion in living bacteria. *Phys Chem Chem Phys* **16**, 12625–12634 (2014).
58. Ershov, D. *et al.* TrackMate 7: integrating state-of-the-art segmentation algorithms into tracking pipelines. *Nat. Methods* **19**, 829–832 (2022).
59. Baym, M., Shaket, L., Anzai, I. A., Adesina, O. & Barstow, B. Rapid construction of a whole-genome transposon insertion collection for *Shewanella oneidensis* by Knockout Sudoku. *Nat. Commun.* **7**, 13270 (2016).
60. Hirose, A. *et al.* Electrochemically active bacteria sense electrode potentials for regulating catabolic pathways. *Nat. Commun.* **9**, 1083 (2018).
61. Firer-Sherwood, M., Pulcu, G. S. & Elliott, S. J. Electrochemical interrogations of the Mtr cytochromes from *Shewanella*: opening a potential window. *J. Biol. Inorg. Chem.* **13**, 849–854 (2008).
62. Marritt, S. J. *et al.* The roles of CymA in support of the respiratory flexibility of *Shewanella oneidensis* MR-1. *Biochem. Soc. Trans.* **40**, 1217–1221 (2012).
63. Pitts, K. E. *et al.* Characterization of the *Shewanella oneidensis* MR-1 Decaheme Cytochrome MtrA. *J. Biol. Chem.* **278**, 27758–27765 (2003).
64. Hartshorne, R. S. *et al.* Characterization of *Shewanella oneidensis* MtrC: a cell-surface decaheme cytochrome involved in respiratory electron transport to extracellular electron acceptors. *J. Biol. Inorg. Chem.* **12**, 1083–1094 (2007).
65. Fonseca, B. M. *et al.* The tetraheme cytochrome from *Shewanella oneidensis* MR-1 shows thermodynamic bias for functional specificity of the hemes. *J. Biol. Inorg. Chem.* **14**, 375–385 (2009).
66. Pessanha, M. *et al.* Tuning of functional heme reduction potentials in *Shewanella* fumarate reductases. *Biochim. Biophys. Acta* **1787**, 113–120 (2009).
67. Xin Jin *et al.* Membraneless organelles formed by liquid-liquid phase separation increase bacterial fitness. *Sci. Adv.* **7**, eabh2929 (2021).
68. Ross, D. E., Flynn, J. M., Baron, D. B., Gralnick, J. A. & Bond, D. R. Towards electrosynthesis in *Shewanella*: energetics of reversing the Mtr pathway for reductive metabolism. *PLoS ONE* **6**, e16649 (2011).
