# Supplementary material for: Spatiotemporal organization of membrane protein controls bacterial extracellular electron transfer
Source: Nat Commun. 2026 Feb 17;17:2855. doi: 10.1038/s41467-026-69655-y (PMC13021941; doi:10.1038/s41467-026-69655-y)
Supplement: Supplementary file 2 — Description of Additional Supplementary File [file 41467_2026_69655_MOESM2_ESM.pdf]

The Description of Additional Supplementary Files

Supplementary Code 1: These Matlab codes are suited for the analysis of single molecule imaging, which was carried out in the research paper entitled " Spatiotemporal organization of membrane protein controls bacterial extracellular electron transfer.
