# Supplementary material for: Spatiotemporal organization of membrane protein controls bacterial extracellular electron transfer
Source: Nat Commun. 2026 Feb 17;17:2855. doi: 10.1038/s41467-026-69655-y (PMC13021941; doi:10.1038/s41467-026-69655-y)
Supplement: Supplementary file 3 — Supplementary Code [file 41467_2026_69655_MOESM3_ESM.zip › SupplementaryCode1/ReadMe.docx]

Introduction for **Supplementary Code 1**

This document describes the MATLAB codes and data analysis procedure for the manuscript “**Spatiotemporal organization of membrane protein controls bacterial extracellular electron transfer**” by Youngchan Park,…Peng Chen *et al.*

The zip file includes 8 folders and a ReadMe.pdf file. The folders include MATLAB codes operated on MATLAB R2023a. Please run the MATLAB codes in the order specified below. The code includes instructions for the procedure.

1. For wide-field fluorescence image analysis

These codes are modified based on iQPALM^1,2^ (Image-based Quantitative Photo-Activated Localization Microscopy) and ITCDD^3^ (inverse transform of confined displacement distribution), reported previously.

1. Folder name [iQPALM_modified] 🡪 for single-molecule tracking analysis and protein quantification
2. Run ‘iQpalm_mknewfd.m’ to make folders to separate multiple raw image files
3. Run ‘Rawdataanalysis2_mac_yp.m’ for raw data analysis

Output: ‘imgshift.mat’ and ‘postfit.mat’

1. Run ‘dataanalysis_mac_yp.m’ for combining multiple ‘imgshift.mat’ and ‘postfit.mat’ files.
2. Run ‘afterdataanalyses_mac_yp’ for fitting the results and plotting.

Output: ‘afterdataanalysis.m’

1. Folder name [ITCDD] 🡪 To deconvolute the cell confinement effect
2. Run ‘CEM2Dmembgeneration.m’ for generating simulated tracks
3. Run ‘ITCDD_result.m’ for fitting the results and plotting
4. Super-resolution reconstructed image
5. Folder name [SuperResImage] 🡪 reconstruction of super-resolution image from single-molecule tracking results
6. Run ‘yp_singlecellsuperesolution.m’
7. For electrochemical fluorescence imaging for real-time monitoring
8. Folder name [ElectroChemImaging] 🡪 image analysis of condensate formation at a single-cell level on electrochemical setup
9. Run ‘yp_DIC_masking_forECpalm.m’ to get cell mask from multiple transmission images

Output: ‘CombinedMask.mat’

1. Run ‘yp_skewness_cal.m’ for skewness calculation for each cell

Output: 'skewcell.mat'

1. Run ‘yp_plot_skewness’ for plotting skewness vs. time with a sigmoidal function fitting
2. Condensate determination by Machine Learning software (ilastik)
3. Folder name [CondensatePickAfterML]🡪 analyze the condensate from binary image obtained from ‘ilastik’
4. Run ‘modifying_granules.m’ to determine which cell contains condensate and position/copy number of condensate.

Output: ‘Filename_Condensate.mat’

1. Analysis of double-tagged strain
2. Folder name [DoubleTaggedProteinQuant] 🡪 analyzed FP double-tagged strain
3. Run ‘ypmakfold_DoubleTagged.m’ to make folders to separate multiple raw image files
4. Run ‘ypDoubleTagged.m’ to get the cell mask from multiple transmission image and analyze each fluorescence color and combine two information

Output: ‘Filename_iQ_coloc.mat'

1. Run ‘DoubleTagged_IntSkew.m’ for copy number quantification and skewness calculation
2. Run ‘ML_DoubleTagged.m’ for condensate analysis with machine learning (ilastick)

Output: ‘ML_Filename.mat’

1. Run ‘ML_Plot.m’
2. For kinetic analysis
3. Folder name [Kinetics] 🡪 for estimation of rate constant of escaping and condensation.
4. Run ‘yp_kinetics.m’
5. For integrated photoelectrochemical current measurement and fluorescence imaging

This code is modified based on previous stduy^4^.

1. Folder name [PhotocurrentFLImag] 🡪 for analyzing fluorescence image
2. Run ‘extract_cell_pos.m’ to get the cell position that we measured the current
3. Run ‘pick_indv_cell.m’ for obtaining each cell information (position, geometry) in order of cells we measured the current

Output: ‘individual_cell_cluster_info.mat’

1. Run ‘ProteinQuant.m’ to quantify protein concentration of each cell

Output: ‘postanal_cellinfo.mat’

1. Run ‘auto_photocurr2.m’ to extract photocurrent

**References**

1. Chen, T.-Y. *et al.* Concentration- and chromosome-organization-dependent regulator unbinding from DNA for transcription regulation in living cells. *Nat Commun* **6**, 7445 (2015).

2. Chen, P. & Chen, T.-Y. MATLAB code package: iQPALM (image-based quantitative photo- activated localization microscopy). https://doi.org/10.6084/m9.figshare.12642617.v1. (2020).

3. Chen, T.-Y. *et al.* Quantifying multistate cytoplasmic molecular diffusion in bacterial cells via inverse transform of confined displacement distribution. *J. Phys. Chem. B* **119**, 14451–14459 (2015).

4. Fu, B. *et al.* Single-cell multimodal imaging uncovers energy conversion pathways in biohybrids. *Nat. Chem.* **15**, 1400–1407 (2023).
