# Supplementary figures and images for: Spatiotemporal organization of membrane protein controls bacterial extracellular electron transfer
Source: Nat Commun. 2026 Feb 17;17:2855. doi: 10.1038/s41467-026-69655-y (PMC13021941; doi:10.1038/s41467-026-69655-y)

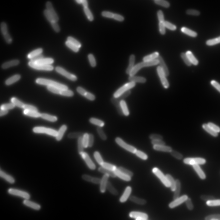

Supplement: Supplementary file 3 — Supplementary Code [file 41467_2026_69655_MOESM3_ESM.zip › SupplementaryCode1/A1_iQPALM_modified/example/Bing_PALM_tracking_1_Bing_SCQPC_561_4ms(3).tif]

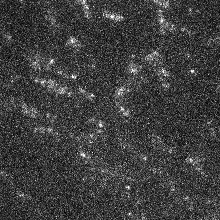

Supplement: Supplementary file 3 — Supplementary Code [file 41467_2026_69655_MOESM3_ESM.zip › SupplementaryCode1/A1_iQPALM_modified/example/Bing_PALM_tracking_1_Bing_Tracking_561_4ms(0).tif]

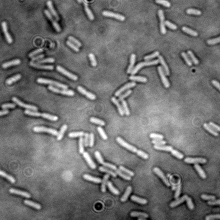

Supplement: Supplementary file 3 — Supplementary Code [file 41467_2026_69655_MOESM3_ESM.zip › SupplementaryCode1/A1_iQPALM_modified/example/DIC1.tif]
